# *De novo* Generation of Cells within Human Nurse Macrophages and Consequences following HIV-1 Infection

**DOI:** 10.1371/journal.pone.0040139

**Published:** 2012-07-18

**Authors:** Suzanne Gartner, Yiling Liu, Senthilkumar Natesan

**Affiliations:** 1 Institute of Human Virology, University of Maryland School of Medicine, Baltimore, Maryland, United States of America; 2 Department of Neurology, Johns Hopkins University School of Medicine, Baltimore, Maryland, United States of America; University Hospital Zurich, Switzerland

## Abstract

Nurse cells are defined as those that provide for the development of other cells. We report here, that *in vitro,* human monocyte-derived macrophages can behave as nurse cells with functional capabilities that include *de novo* generation of CD4+ T-lymphocytes and a previously unknown small cell with monocytoid characteristics. We named these novel cells “self-renewing monocytoid cells” (SRMC), because they could develop into nurse macrophages that produced another generation of SRMC. SRMC were not detectable in blood. Their transition to nurse behavior was characterized by expression of CD10, a marker of thymic epithelium and bone marrow stroma, typically absent on macrophages. Bromodeoxyuridine labeling and immunostaining for cdc6 expression confirmed DNA synthesis within nurse macrophages. T-cell excision circles were detected in macrophages, along with expression of pre-T-cell receptor alpha and recombination activating gene 1, suggesting that genetic recombination events associated with generation of the T-cell receptor were occurring in these cells. SRMC expressed CCR5, the coreceptor for R5 HIV-1 isolates, and were highly susceptible to HIV-1 entry leading to productive infection. While expressing HIV-1, SRMC could differentiate into nurse macrophages that produced another generation of HIV-1-expressing SRMC. The infected nurse macrophage/SRMC cycle could continue *in vitro* for multiple generations, suggesting it might represent a mechanism whereby HIV-1 can maintain persistence *in vivo*. HIV-1 infection of nurse macrophages led to a decline in CD4+ T-cell production. There was severe, preferential loss of the CCR5+ CD4+ T-cell subpopulation. Confocal microscopy revealed individual HIV-1-expressing nurse macrophages simultaneously producing both HIV-1-expressing SRMC and non-expressing CD3+ cells, suggesting that nurse macrophages might be a source of latently infected CD4+ T-cells. Real-time PCR experiments confirmed this by demonstrating 10-fold more HIV-1-genome-harboring T-cells, than virus-expressing ones. These phenomena have far-reaching implications, and elicit new perspectives regarding HIV pathogenesis and T-cell and hematopoietic cell development.

## Introduction

**Figure 1:**
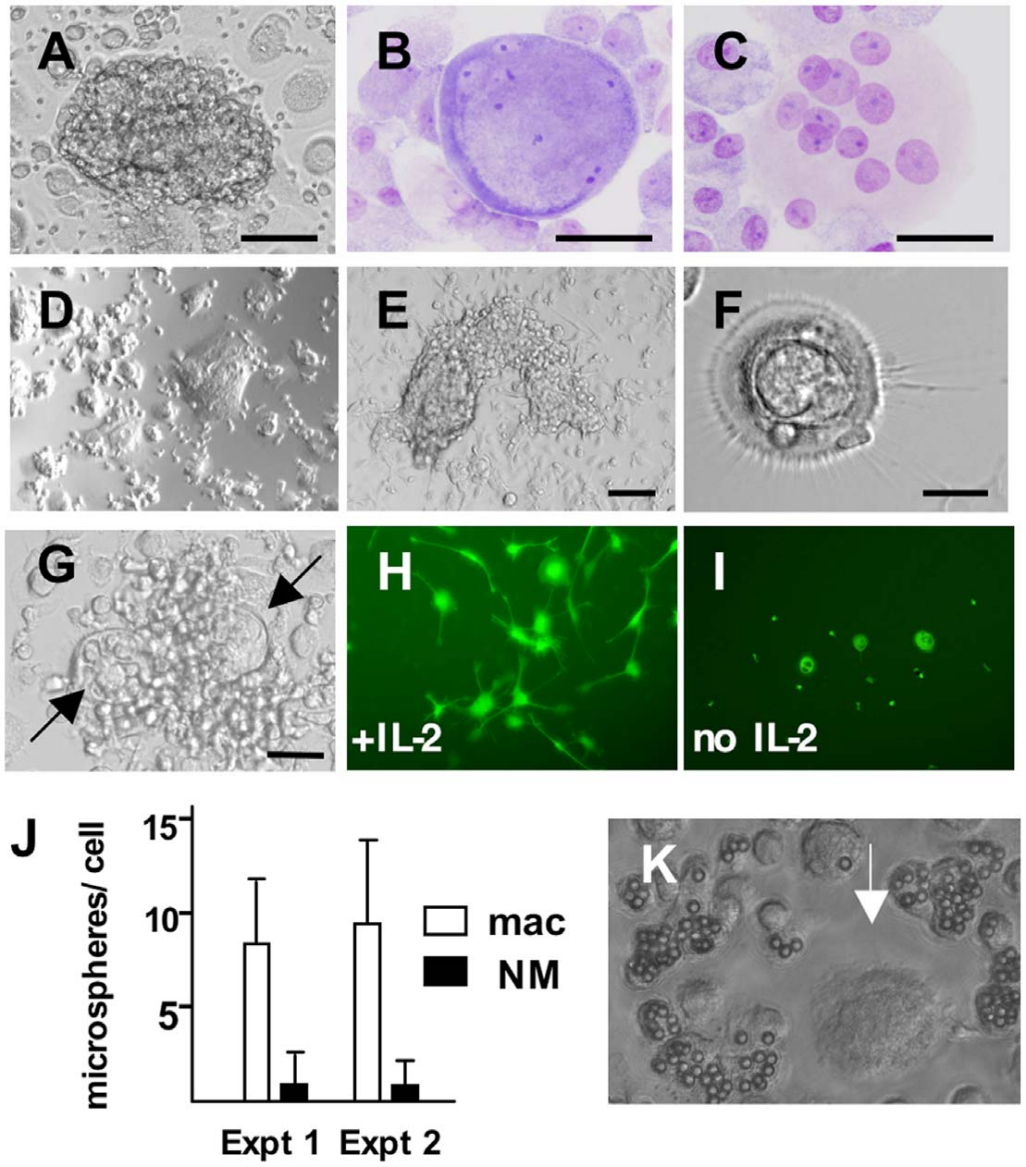
Human macrophage phenotypes. (A) complex colony in MDM culture. (B, C) Wright’s-Giemsa-stained, large multinucleated cells, with atypical (B) and typical (C) morphology, recovered from the adherent population of a 31-day-old MDM culture. (D-G) Responses of EDTA-recovered replated macrophages to IL-2. Macrophages were detached on D31 of culture, replated at low density, and IL-2 added 2 days later, after extensive washing. Cells were photographed on day 8 (D-F) or day 6 (G) of IL-2 exposure. Arrows in (G) point to nurse macrophages within a cell cluster. (H, I) IL-2-mediated induction of dendritic processes on replated macrophages. To aid visualization, shown here are macrophages infected with an HIV-1 strain harboring the enhanced green fluorescent protein gene. Macrophages were infected with HIV-1 pSV162R3, subjected to EDTA removal 15 days following infection, and replated. IL-2 was added 4 days later, after washing, and the treated and untreated cultures photographed on day 5. Similar responses of uninfected cells to IL-2 are shown in Fig. S1. (J, K) Nurse macrophages exhibit reduced phagocytic capabilities. Macrophage cultures were established directly from PBMC in 24-well plates. (No Il-2 was used.) To assess phagocytosis, the cells were incubated with 6 mm plastic microspheres for 24 hours, then washed to remove excess spheres and scored. For scoring, NM were identified by size, morphology and the presence of multiple nuclei. The fluorescent dye Hoechst 33342 was added to visualize cell nuclei. (J) Quantitation of microspheres per cell. Fields were selected for scoring based on the presence of NM; 100 macrophages of each type were scored per experiment. Error bars depict SEM. Similar results were obtained in two additional experiments. (K) Apparent is a large NM lacking microspheres (arrow), surrounded by several microsphere-containing macrophages. Size bars: (A) and (E) 100 µm, (B) (C) and (F) 25 µm and (G) 50 µm. Magnification: (D) and (I) x100, (H) x200 and (K) x400.

For years, we noticed the presence of complex, colony-like structures in long-term cultures of human blood monocyte-derived macrophages (MDM) that resembled embryoid bodies and neurospheres (Fig. 1A). These structures were most abundant along the edges of culture vessels and in HIV-1-infected cultures, they corresponded to areas rich with virus-expressing multinucleated giant cells. In young uninfected cultures, the structures contained hundreds of tightly packed cells of varying sizes, including many cells smaller than resting lymphocytes. In older cultures, the structures receded and these areas became densely populated with maturing macrophages. During experiments aimed at dissociation of the colonies in uninfected cultures, we recovered large, viable, multinucleated adherent cells with unique morphologies. These observations were the impetus for studies reported here. We found that human MDM can behave as nurse cells with functional capabilities that include the development and release of newly formed cells, a previously unknown phenomenon.

Cells that support the development of other cells are often referred to as nurse cells, for example, the ovarian nurse cells of *D. melanogaster* that play a pivotal role in early oogenesis [1]. In 1980, Wekerle and Ketelsen coined the term “thymic nurse cell” to describe large, Keratin+ Ia+ epithelial cells recovered from mouse thymus that contained lymphocytes enclosed within membrane-lined caveoli [2]. Human thymic nurse cells have also been described [3], but debate continues as to whether these structures correspond to sites of T-cell development, or represent artifact. There is little disagreement, however, that thymic epithelial cells are key participants in intrathymic T-cell development. Although Wekerle and Ketelsen found that thymic nurse cells and macrophages had identical patterns of surface antigen expression, they concluded that thymic nurse cells were not macrophages because they lacked phagocytic activity and behaved more like epithelial cells [2], [4]. Reduced phagocytic activity and an epitheloid appearance are characteristics of epithelioid histiocytes [5], a macrophage-derived, primary constituent of granulomas. The existence of these cells suggests a phenotypic and functional continuum between the macrophage and certain epithelial cells found in some anatomical locations.

Macrophage lineage cells manifesting nurse cell function have also been described. For example, erythroid cell development in bone marrow, which occurs within structures termed erythroblastic islands, is dependent upon a central “nurse” macrophage, which is thought to provide nutrients and proliferative and survival signals to the erythroblasts [6]. Also, an adherent population derived from CD14+ blood cells has been described as “nurse-like” based on its ability to prevent apoptosis in chronic lymphocytic leukemia B-lymphocytes [7], [8]. The reported capabilities of these nurse-like macrophages did not include the generation of new cells. However, the continuous generation and release of monocytoid cells by avian monocyte-derived multinucleated osteoclasts has been reported [9].

While HIV-1 infection of macrophage lineage cells is well documented [10]–[12], many basic features of this infection remain unclear. In particular, at what stages of maturation are the cells susceptible to viral entry? Also, are macrophages hosts of significance for latent infection *in vivo,* and how much do they contribute to viral persistence? It is clear that despite years of uninterrupted highly active antiretroviral therapy (HAART), HIV-1 persists within the body. This persistence has been linked to the presence of a reservoir of latently infected CD4+ T-lymphocytes (CD4T) [13], [14], in particular, aged memory CD4T (CD45RO+CD57+) [15]. Stabilization at “set” levels of plasma viremia can occur after 1–2 years on HAART [16], [17], but there is debate as to how much of this viremia is attributable to incomplete suppression of ongoing virus replication, reactivation of latently infected cells [18], or intermittent production from stable reservoirs like macrophages. Because drug resistance and viral evolution do not typically characterize the residual HIV-1 strains seen in patients with persisting low-level viremia [19], [20], ongoing replication seems a less likely possibility, unless the anatomical locations of residual replication strongly prohibit drug penetration. Withdrawal of HAART, even after long periods, leads to rebound viremia within days or weeks, and the rebounding viral strains often lack antiretroviral resistance mutations. Also, the intermittent viremia that occurs in patients on HAART is not associated with acquisition of new resistance mutations before, during or after the viremia [21].

Studies of SIV infection in macaques first brought to the forefront the importance of mucosal tissue in acute and chronic infection [22], [23]. An analogous situation also characterizes HIV-1 infection [24], [25]. It is now understood that mucosal tissues are not only the principal sites of viral transmission, but also the primary sites of virus replication and CD4T depletion [23], [25], [26]. Underlying these occurrences are the facts that (1) mucosal tissues contain a much greater abundance of CCR5-expressing CD4T than blood or lymph nodes, and (2) this subpopulation is the preferred target for infection [25], [27]. Also, the progressive CD4T depletion that underpins much of HIV/SIV clinical disease, is attributable primarily to loss of CCR5+ CD4T, a disease association strengthened by the findings that (1) natural hosts for these viruses exhibit strikingly reduced levels of CCR5 on CD4T [28], and (2) the CCR5Δ32 mutation confers resistance to HIV-1 [29]. Along with chemokine coreceptor expression, the state of cellular activation at the time of viral entry is also a critical determinant of whether productive infection is established [30], [31]. It has further been shown that gut-associated lymphoid tissue (GALT) can represent a major site for persistence of infection in individuals receiving effective antiretroviral therapy for years and moreover, it has also been shown that cross-infection between GALT and blood occurs, which could account for the continued presence of circulating infected cells [32]. Several mechanisms have been proposed to account for the continuing decline of CD4T in the natural history setting, including direct killing mediated by virus infection, indirect killing (bystander effects), and chronic immune activation. We show here that *in vitro*, nurse macrophages are a primary source of CCR5+ CD4T, and that their infection with HIV-1 results in a dramatic and specific loss of this CD4T subpopulation. These findings may recapitulate events that occur *in vivo*, particularly within gut mucosa, and help to explain the CD4T decline in HIV/AIDS.

Thus, in this report, we describe a highly significant and hitherto unrecognized behavior of the human macrophage, which HIV-1 exploits for its survival. Integral to this survival strategy is infection of a novel cell, the self-renewing monocytoid cell (SRMC), which develops within and is released from a macrophage, and is renewed via cycles of SRMC/nurse macrophage development. HIV-1 has positioned itself to be carried along with this process.

## Results

### Development of T-cell-producing Nurse Macrophages

Long-term cultured human macrophages cannot be removed readily from culture vessels using traditional methods such as treatment with trypsin. Using 4- to 6-week-old MDM cultures, we performed experiments to determine conditions optimal for colony disruption and macrophage detachment and found that a significant portion of these cells detach following exposure to 20 mM EDTA for 10–15 minutes. The cells recovered were highly viable, of variable size, and capable of being replated. Among those recovered were large (≥50 µm), viable, multinucleated single cells. Some had indistinct nuclear/cytoplasmic borders, rims of densely stained material, and an overall appearance reminiscent of thymic nurse cells (Fig. 1B). They were distinguishable from more typical multinucleated macrophages that have well-defined nuclei and faint cytoplasm (Fig. 1C). Owing to this nurse cell appearance, Interleukin-2 (IL-2) was added to replated adherent cells seeded at low density (6×10^4^–2.6×10^5^/well in 24-well plates). Prior to addition of IL-2, replated cells were cultured for 2–4 days, and then washed extensively to remove all nonadherent cells. The IL-2-treated cells established a network-like microenvironment that ultimately included giant epithelioid cells (Fig. 1D) and complex, colony-like structures (Fig. 1E). Large round stellate macrophages with a distinct morphology characterized by an inner ring-like structure and small round cells enclosed within, also appeared (Fig. 1F). With time, many large macrophages harboring small cells, internally, became apparent; we named these “nurse macrophages” (NM). Small cells budded from NM and colonies developed around them (Fig. 1G). Both nonadherent cells and small adherent cells were released; nonadherent cells expanded with time. Initiation of the microenvironment, which was apparent as early as day 2 of IL-2 exposure, was characterized by development of long, thin bi- and multi-polar processes (Fig. 1H and I and Fig. S1). Process development was more common among the smaller adherent cells. We further confirmed the macrophage derivation of these phenomena using CD14+ cells purified from peripheral blood mononuclear cells (PBMC) (Fig. S2). Also, NM exhibited reduced phagocytic capabilities (Fig. 1 J and K).

**Figure 2:**
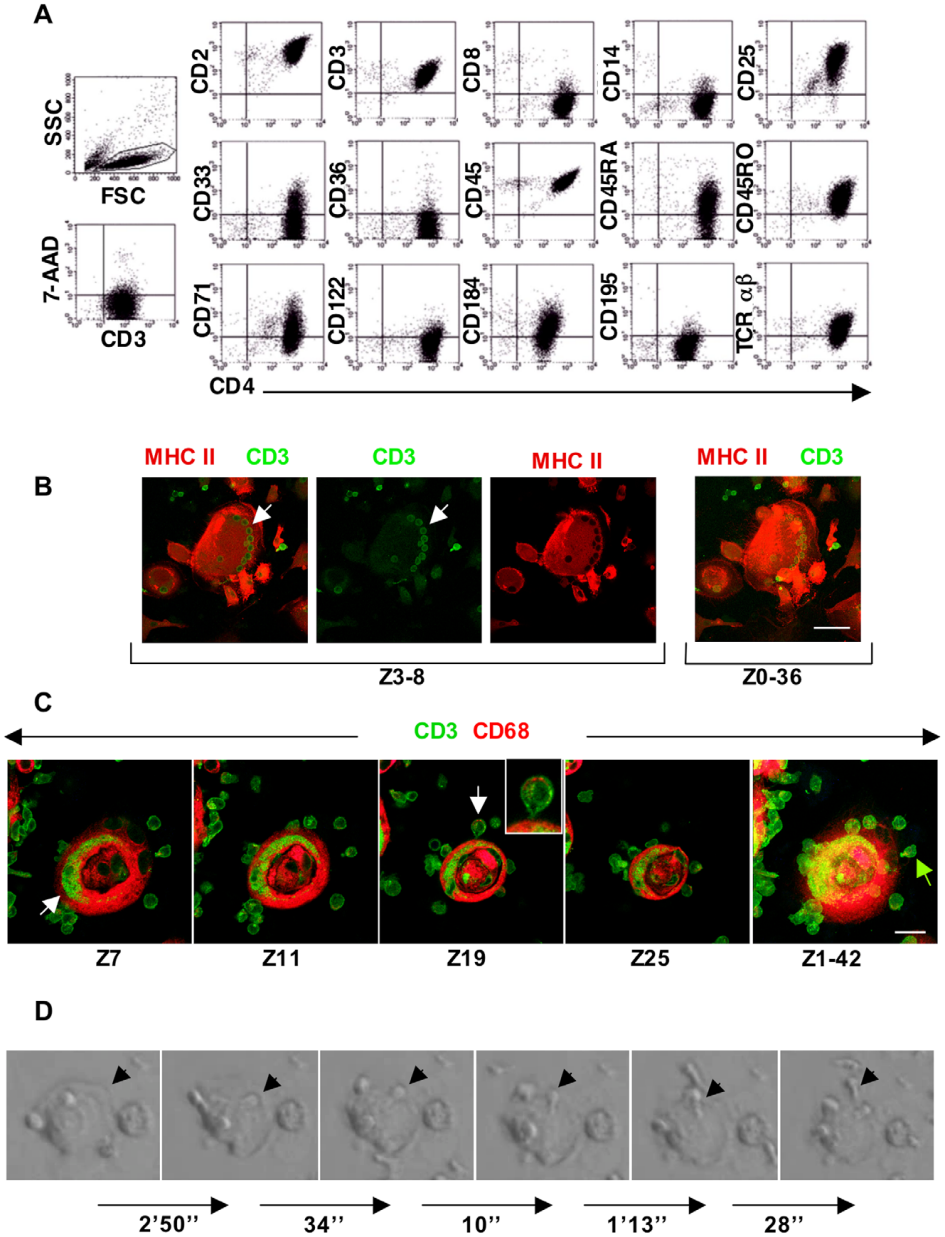
T-cells are produced within nurse macrophages. (A) Immunophenotype of nonadherent cells harvested from cultures of replated EDTA-recovered macrophages after 112 hours of exposure to IL-2. The forward (FSC) versus side (SSC) scatter plot shown on the left indicates the gate used for the other plots. As can be seen, the vast majority of cells are CD4+ T-lymphocytes. The 7-AAD plot indicates that these cells were viable. (B) Confocal microscopy images showing that the CD3+ cells present within this large MHC II+ macrophage appear as an organized array. This culture of replated EDTA-recovered macrophages was photographed on day 10 of exposure to IL-2. Size bar: 50 µm. (C) Confocal microscopy images illustrating CD3+ cells within a CD68+ macrophage at day 10 of IL-2 exposure. Free CD3 antigen deposition is also apparent within the macrophage (white arrow, far left panel), as is budding of a CD3+ cell from the macrophage surface (green arrow, far right panel). Boxed insert in the center panel is an enlargement of the cell identified by the white arrow. Small pockets of CD68 antigen can be seen within this CD3+ cell near the top edge. Size bar: 20 µm. Z indicates the Z-stack level(s) for each image. See Fig. S4 for a gallery view of this series. (D) Time-lapse video images of a lymphocyte budding from a macrophage in a replated culture exposed to IL-2 for 7 days. (The budding cell is a lymphocyte, based on its subsequent nonadherent post-bud behavior. Data not shown.) Numbers below the arrows refer to the time interval in minutes and/or seconds between the two images. Arrowheads identify the position of the budding cell, which first becomes apparent in the second panel.

Flow cytometry was used to characterize the nonadherent cells produced in EDTA-recovered IL-2-treated macrophage cultures (EDTA/IL2-mac). Cells were harvested at regular intervals, beginning with their earliest appearance at 2–4 days of IL-2 exposure. Peak production occurred between days 7–16 of exposure, when 1–2×10^6^ viable nonadherent cells were recovered per well of 24-well plates every 3–4 days. Immunophenotyping was performed at each harvest. An example of the results is shown in Fig. 2A. The primary population, which represented >90% of cells within the lymphocyte gate, was always CD3+CD4+. These cells expressed the T-cell markers CD2, CD3 and CD4, but not CD8. They were negative for markers of B-cells (CD19), natural killer (NK) cells (CD56) and stem cells (CD34, CD117 and CD133). (Negative data not shown.) Throughout the culture period, all CD4+ T-cells expressed CD45RO and a portion coexpressed CD45RA. The nonadherent population contained small numbers (<1.0%) of other cells, including CD8+ and CD4+CD8+ T-cells, and CD34+ cells (Fig. S3). Similar results were obtained from 15 independent experiments using cells from 7 normal donors. We emphasize that the replated cells used in these experiments were recovered from primary macrophage cultures of at least 4 weeks of age, and reseeded at low density.

While the budding of small cells from large macrophages, particularly from those with a nurse-like morphology, could readily be seen in living cultures, we used confocal microscopy to confirm and further elucidate this process. Confocal microscopy using CD3 as a T-cell marker and CD68 or major histocompatibility complex (MHC) class II as a macrophage marker, revealed that the large multinucleated macrophages in EDTA/IL2-mac cultures housed CD3+ cells. Within individual NM, organized arrays of CD3+ cells were observed (Fig. 2B white arrow), as well as discreet regions of CD3 antigen (Fig. 2C left panel, white arrow; gallery view in Fig. S4). The developing lymphocytes appeared located within caveoli-like structures. They exited NM by budding from the cell or being released at the cell surface (Fig. 2C right panel, green arrow, and Fig. 2D). Small pockets of CD68 antigen were visible within some post-budded T-cells (Fig. 2C center panel, white arrow). These cells were located closely adjacent to, or still attached to, a NM, suggesting that they were newly formed. This could reflect incorporation of small amounts of this macrophage antigen into the cytoplasm of the T-cells as they were formed and released. Residual T-cells would not be expected to harbor CD68, and we have not observed CD68 immunoreactivity in T-cells cultured under traditional conditions. CD8+ cells were not detected budding from NM, nor was CD8 antigen detected within.

**Figure 3:**
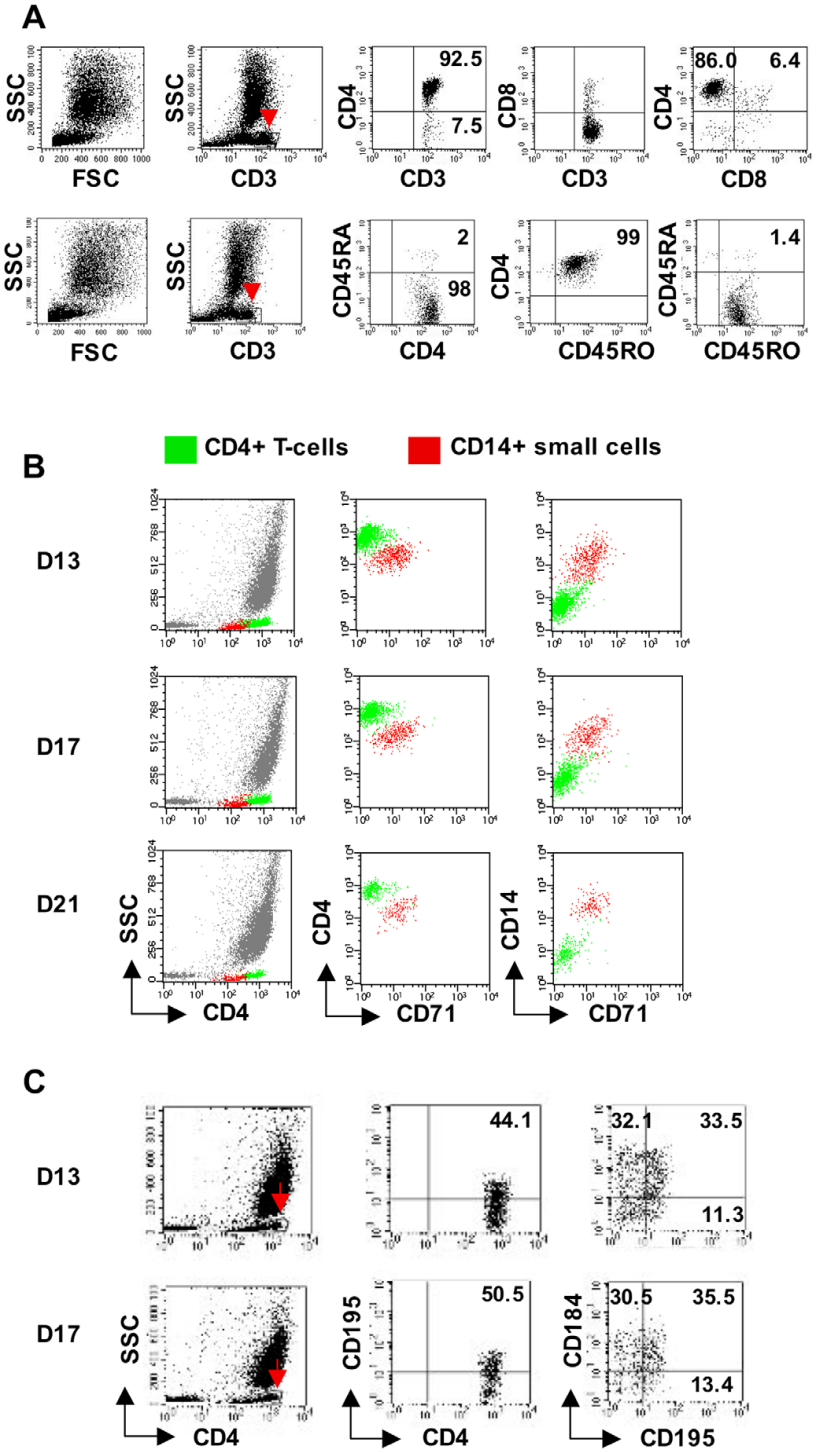
Characteristics distinguishing CD4+ T-cells produced in primary macrophage cultures from those produced in EDTA/IL2-mac. (A) As was the case in cultures of replated EDTA-mac treated with IL-2, the vast majority of T-cells produced in primary MDM cultures were also CD4+ T-cells. This is depicted here. However, in contrast to IL-2 treated cultures, these CD4+ T-cells were almost exclusively CD45RO+ and lacked CD45RA. Cells shown here were harvested on day 13 of culture and gated based on small size and CD3 expression (red arrows). The forward versus side scatter plots on the left demonstrate the relative numbers of large macrophages and lymphocytes within the nonadherent cell population. The numbers shown within the quadrants represent percentages from among the gated populations. (B) In contrast to those recovered from EDTA/IL2-mac, as shown here, the CD4+ T-cells produced in primary macrophage cultures did not express CD71, the transferrin receptor, indicating that they were resting lymphocytes. The CD71+ cells apparent are small CD4dim/CD14+ monocytoid cells, not lymphocytes. Analyses performed using Paint-a-Gate software. (C) Also in contrast to the CD4+ T-cells produced in IL-2-treated cultures, as shown here, 40–50% of these cells produced in primary MDM cultures expressed CD195, and 30–35% were dual positive for CD195 and CD184. Gates for the dot plots shown were established based on CD4bright expression and low side scatter, as indicated in the left panels (red arrows). Small, CD4dim cells were excluded from the gates because these were small monocytoid cells, not T-cells. The numbers shown within the quadrants represent percentages from among the gated populations. Analyses performed using CellQuest software. For (B) and (C), the letter D refers to the age of the culture in days at the time of harvest.

Having observed that our primary long-term MDM cultures are characterized by persistence of (1) adherent cells with nurse morphology and (2) highly viable nonadherent cells that establish new macrophage cultures when replated, we sought to determine if T-cell production was ongoing in these primary cultures in the absence of IL-2 or any other exogenous growth factors. (We emphasize that these cultures are distinct from EDTA/IL2-mac. They are primary, not secondary cultures, and no IL-2 is added.) Again, flow cytometry was used to characterize the nonadherent cells that appeared over time, and confocal microscopy employed to examine the adherent macrophages. The number of nonadherent cells recovered averaged 1–2×10^6^ per T25 flask at day 7–14, 5–10×10^5^/T25 at day 18–25 and 0.5–1×10^5^/T25 at day 27–42. Low-level production was still apparent even at day 50–60. While most nonadherent cells were macrophages, CD4T were also significantly represented (Table 1). Some differences between these CD4T and those recovered from EDTA/IL2-mac were apparent. As shown in Figure 3A, almost all of those recovered from the primary macrophage cultures were exclusively CD45RO+. Also, these T-cells failed to express CD71, indicating lack of activation (Fig 3B), and in contrast to those recovered from EDTA/IL2-mac, both CD195(CCR5)+ and CD184(CXCR4)+CD195+ CD4T were represented (Fig. 3C).

**Table 1:** Characteristics of nonadherent cells in primary MDM cultures.

Days of culture	CD4+ T-cells	2+3+16+32+	Small monocytoid cells	MM^c^	CD10+ MM
	(% of NAC^a^)	(% of CD3+)	(% of small cells^b^)	(% of NAC)	(% of MM)
4	27.6	not detected	not detected	9.3	not detected
7	25.0	0.3	not detected	12.5	1.4
11	6.7	3.2	2.2	82.4	5.2
14	8.4	12.0	6.0	79.6	8.6
18	6.6	10.8	9.9	81.7	13.1
21	10.9	6.5	4.4	80.4	not determined

a NAC, nonadherent cells;

b Small cells defined as those within the lymphocyte gate. Small monocytoid cells identified by size and the phenotype presented in Fig. 5A.

c MM, maturing macrophages, defined as large CD14+CD33+ cells with high forward and side scatter. Days 4 and 7 include residual PBMC. Remaining cells from harvested populations include CD8+ T-cells and B, NK and stem cells. Values shown are the percentages determined by flow cytometry for nonadherent cells from pools of six T-25 flasks.

CD4+ T-cells were generated by NM in primary MDM cultures in the absence of exogenous IL-2, and in cultures of replated EDTA/IL2-mac. Exogenous IL-2 accelerated and enhanced development of a microenvironment that supported T-cell development. It also maintained proliferation of post-budded T-cells, which did not occur in the primary MDM cultures.

### Fc Receptor Expression Distinguishes T-cells Derived from Nurse Macrophages

**Figure 4:**
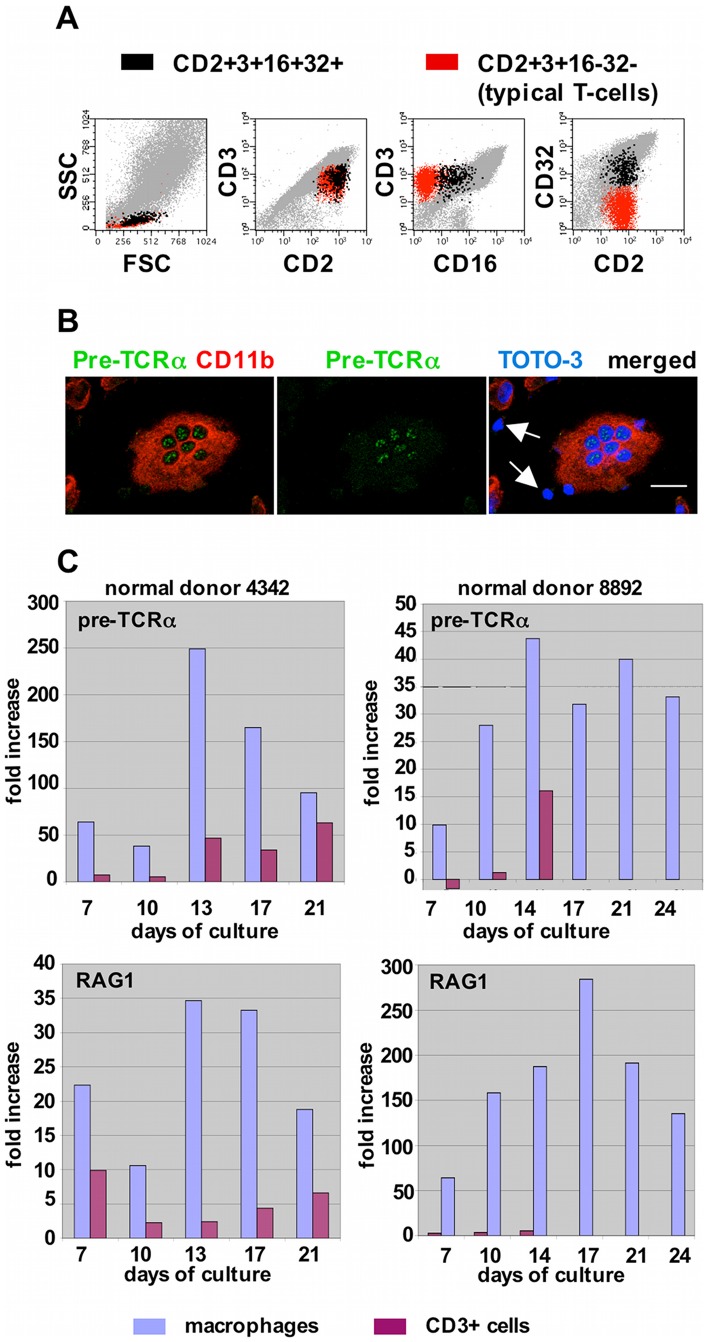
Markers of T-cell immaturity in nurse macrophage-derived T-cells and within nurse macrophages. (A) Detection of CD16+CD32+ T-cells among the nonadherent population harvested at day 21 from primary MDM cultures. At this time, the CD16+CD32+ population represented 6.5% of the total CD3+ cells (also see Table 1). (B) Expression of pre-TCRα associated with nuclei within a nurse macrophage from a 13-day-old primary culture. (Primary cultures do not contain IL-2.) Arrows in the right panel indicate CD11b-negative cells, presumed to be T-cells, not expressing pre-TCRα. Size bar: 20 µm. (C) Expression of pre-TCRα and RAG1 in macrophages and CD3+ cells recovered from primary macrophage cultures, as determined by real-time PCR. CD3+ cells were purified from the nonadherent populations using CD3+ magnetic beads. Macrophages were harvested using accutase. GAPDH was used as a housekeeping gene to normalize values. For each donor, the mean values obtained from uncultured CD3+ cells were subtracted from the mean values shown for CD3+ cells at each time point, and similarly, the mean values obtained from uncultured CD14+ cells were subtracted from the mean values obtained for the cultured macrophages at each time point. No values are shown for CD3+ cells for donor 8892 at days 17, 21 and 24, because too few nonadherent cells were recovered to permit CD3+ magnetic bead selection. The average percent difference between replicate reactions was 0.5% (donor 4342) and 1.3% (donor 8892) for pre-TCRα, and 0.9% (donor 4342) and 0.4% (donor 8892) for RAG1.

CD16(FcγRIII) together with CD32(FcγRII) expression defines an early double negative thymocyte in the fetal mouse thymus [33], [34]. Whether this population exists in the human fetal or adult thymus is unknown. We detected increasing numbers of CD2+CD3+CD16+CD32+ cells among the nonadherent population recovered from primary MDM cultures (Table 1 and Fig. 4A). This population typically represented ∼5–10% of the CD3+ cells. It was not detected in uncultured, mitogen-activated, or IL-2 maintained PBMC, suggesting that it does not represent residual T-cells carried over from the seeding PBMC, and then induced to express Fc receptors via cellular activation or some other stimulus. Based on their increasing frequency, but low abundance, we reasoned that these CD2+CD3+CD16+CD32+ cells were T-cells that have recently exited NM and that as they further matured, they lost the Fc receptor molecules. To further identify the properties of these cells, we sorted the CD2+CD3+CD16+CD32+ populations from among nonadherent cells harvested from primary macrophage cultures, and assessed their ability to proliferate in response to mitogen (phytohemagglutinin) or IL-2 alone. The results of these experiments are presented in Fig. S5. The CD2+CD3+CD16+CD32+ cells were able to proliferate in the presence of IL-2 for at least 17 days, but died within 3 days when exposed to mitogen. Flow cytometry confirmed that CD16 and CD32 expression were lost from the sorted cells during their culture with IL-2.

### Detection of Pre-TCRα and RAG1 Expression in Macrophages

T lymphocytes bear either αβ or γδ T-cell receptors (TCR) that lend specificity to antigen recognition. Prior to expression of αβ TCR, developing thymocytes express the pre-TCR, consisting of a TCR β-chain covalently associated with the pre-TCR α-chain [35], [36]. Using confocal microscopy, we detected pre-TCRα expression within NM cultured without IL-2 (Fig. 4B). This expression was associated exclusively with what appeared to be nuclei, as evidenced by TOTO-3 staining. Based on comparison of these cells with those stained with both T-cell and macrophage markers, we reason that these pre-TCRα+ nuclei correspond to developing T-cells. In these experiments, Pre-TCRα expression was not seen in lymphocytes that had exited NM (arrows in Fig. 4B). Similar patterns of expression were seen in EDTA/IL2-mac and in these cultures, there was also occasional expression in post-budded independent CD3+ cells. (Data not shown.) In addition, we used real-time PCR to assess pre-TCRα expression in adherent macrophages and nonadherent CD3+ cells harvested from primary macrophage cultures over time. (No IL-2 was used in these experiments.) As shown in Fig. 4C, pre-TCRα expression was detected in the macrophages at all times examined, and at levels exceeding those detected in the CD3+ cells.

Recombination activating gene 1 (RAG1), and recombination activating gene 2 (RAG2) encode proteins that play essential roles in the genetic recombination events associated with development of the T-cell receptor [37]. We hypothesized that if TCR rearrangement takes place within NM, RAG1 would likely be expressed. We investigated this possibility using real-time PCR, and the specimens used in the pre-Tα expression experiments described above. As shown in Fig. 4C, as was the case for pre-Tα expression, RAG1 expression was detected in macrophages at all times examined, and at levels exceeding those detected in the T-cells (CD3+ cells).

### Detection of δRec-ψJα T-cell Receptor Excision Circles in Macrophage Cultures

During the VDJ recombination events associated with development of the T-cell receptor, fragments of DNA between the rearranging V, D and J gene segments are excised as circular molecules. These molecules are referred to as T-cell receptor excision circles (TREC) [38], [39]. TREC can be found in thymus, as well as peripheral blood, and in blood, their presence is considered a marker of T-cells that have recently left the thymus [40]. TREC numbers in blood are decreased in both pediatric and adult HIV-1-infected individuals, and measurement of TREC has been used to evaluate immune reconstitution following introduction of ARV therapy in HIV/AIDS [41], [42] and also in the setting of hematopoietic stem cell transplant [43]. TREC are episomal and do not replicate, so they are lost (diluted out) during T-cell proliferation. Most commonly, TREC that contain a δRec-ψJα signal joint are the target of detection and quantification [39]. Using real-time PCR and a molecular construct to quantitate copy number, we examined cultured macrophages for the presence of TREC, reasoning that these molecules should necessarily be present if VDJ rearrangement was taking place. As Table 2 demonstrates, δRec-ψJα TREC were detected in adherent cells (macrophages) following exposure to IL-2, with first appearance typically around day 7 of exposure. The window of detection was limited to one week, at most. (We speculate that macrophages might degrade extrachromosomal DNA.) TREC were also detected in the nonadherent population, which primarily consisted of T-cells generated by NM during the culture period, and then further maintained in a proliferative state by the presence of IL-2. Sequencing confirmed the TREC identity of the PCR products (Fig. S6). As Table 2 shows, most often, TREC detection in macrophages preceded that in the nonadherent population (T-cells). Because the CD4+ T-cells produced by NM are loosely adherent for a short while after exit from the mother cell, in two experiments, we independently examined the loosely adherent cells. TREC were detected in these cells, suggesting that the CD4+ T-cells generated within NM could harbor TREC during their release from NM. At this time, we cannot exclude the possibility that the recombination events that yield TREC also took place within nonadherent post-budded T-cells. Our pre-Tα and RAG1 expression data, however, suggest that macrophages were the more frequent hosts for these events.

**Table 2:** Detection of δRec-ψJa T-cell receptor excision circles in macrophage cultures.

Specimen^a^	IL-2 exposure (days)	Cell population^b^	TREC/µg DNA
Donor 225 adherent cells	Pre-exposure	Adherent	0^c^
	4	Adherent	0
	7	Adherent	0
		Nonadherent	0
	8	Adherent	212±120
		Nonadherent	0
	9	Adherent	0
		Nonadherent	0
	11	Adherent	Indeterminate
		Nonadherent	26±14
	14	Adherent	0
		Nonadherent	10±2
	16	Adherent	0
		Nonadherent	0
	18	Adherent	0
		Nonadherent	0
	20	Adherent	0
		Nonadherent	0
Donor 914 adherent cells	Pre-exposure	Adherent	0
	7	Adherent	65±2
		Nonadherent	0
	10	Adherent	39,412±1152
		Nonadherent	0
	12	Adherent	0
		Nonadherent	545±20
	14	Adherent	0
		Nonadherent	0
Donor 898 adherent cells	Pre-exposure	Adherent	0
	7	Adherent	31±11
		Nonadherent	405±69
	10	Adherent	0
		Nonadherent	0
	12	Adherent	0
		Nonadherent	0
Donor 895 CD3-depleted adherent cells	13	Adherent	78±38
		Loosely adherent	65±18
		Nonadherent	Indeterminate
	17	Loosely adherent	26±5
		Nonadherent	0
	19	Adherent	39±3
Donor 898 CD3-depleted adherent cells	13	Adherent	116±59
		Loosely adherent	50±0
		Nonadherent	27±14
	17	Loosely adherent	0
		Nonadherent	0
	19	Adherent	0
Human thymus (pos control)	Uncultured	Unfractionated	∼100,000^d^
U138MG glioblastoma cells (neg control)	None	Cell line	0

a Adherent cells from Donors 914 and 898 were harvested from primary macrophage cultures at day 38 and 42, respectively, using accutase, and replated in 24-well plates. For the donor 225 cultures, nonadherent cells recovered from day 13 primary cultures were plated at low density in T150 flasks, and then thirteen days later, the adherent cells were harvested and replated in 24-well plates. Adherent cells from donors 895 and 898 were harvested from primary cultures at day 26, depleted of CD3+ cells, and then replated in 24-well plates;

b nonadherent cells were recoverable by washing, loosely adherent cells were primarily T-cells that required short-term accutase treatment for detachment, and adherent cells were macrophages;

c the “0” values shown indicate that no TREC were detected;

d this value represents an approximate average value among different assays. The TREC values shown represent the mean of replicate samples ± standard deviation.

### Nurse Macrophages Give Rise to a Novel Small Cell

**Figure 5:**
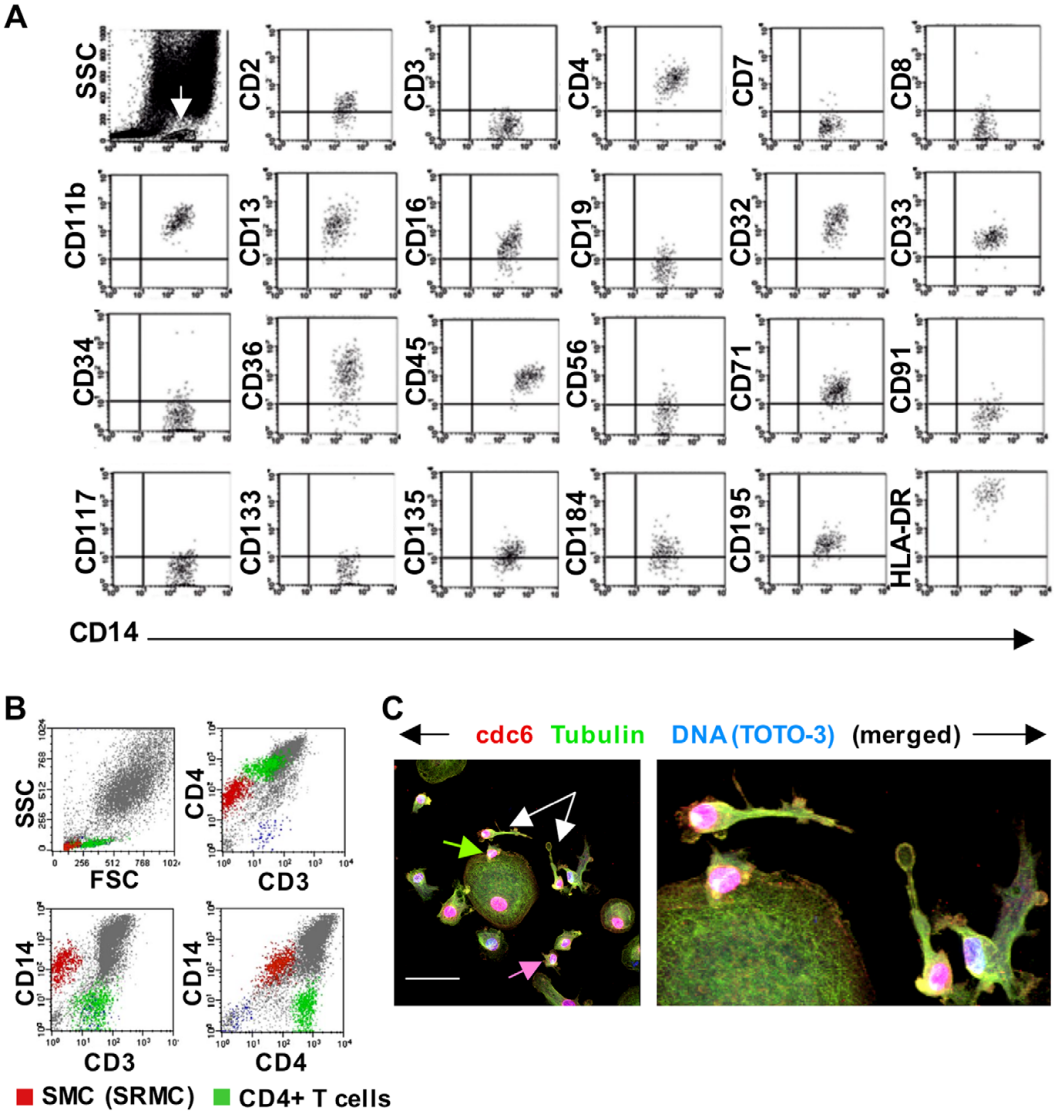
Characteristics of small monocytoid cells produced by nurse macrophages. (A) Immunophenotype of nonadherent small monocytoid cells (SMC) harvested from an 18-day-old MDM culture. White arrow (top left plot) identifies the gated population shown in other plots. (B) Forward scatter (FSC) versus side scatter (SSC) plot demonstrates that SMC (CD3negCD4dimCD14+) are smaller than resting lymphocytes. Cells from 17-day-old MDM. Analysis performed using Paint-a-Gate software. (C) Confocal microscopy demonstrating that SMC adhere following their exit from nurse macrophages. Shown here is an 18-day-old primary MDM culture. An SMC adhering to a large macrophage (green arrow) and another adhering to the culture dish (pink arrow) can be seen. Others with elongated processes are also visible (white arrows) and viewable in greater detail in the panel on the right. The pink color of the nuclei reflects cdc6 colocalization with DNA as identified by TOTO-3 staining. SMC nuclei are typically cdc6+ during and shortly after budding from nurse macrophages, reflecting recent DNA synthesis. Size bar: 50 µm.

The nonadherent cells from primary MDM cultures also included a population of previously uncharacterized small monocytoid cells that did not become detectable until ∼day 11 of culture (Fig. 5 and Table 1). Their immunophenotype is shown in Fig. 5A. They were smaller than lymphocytes (Fig. 5B). Measurements using microscopy and flow cytometry indicated they were ∼6 µm in diameter. They were most abundant at day 13–21 of culture, when they typically represented 4–10% of the total nonadherent population, or 12–33% of cells in the lymphocyte/stem cell gate. Ongoing microscopic examinations and transfer of nonadherent cells to fresh flasks, made clear that these small monocytoid cells constituted a higher percentage of cells produced by NM. Their numbers were reduced owing to their propensity for rapid adherence (Fig. 5C). Microscopic examinations also revealed that these small adherent cells could mature and develop into macrophages, including NM that produced another generation of small monocytoid cells. For this reason, we propose the name “self-renewing monocytoid cells” (SRMC). This population was not detectable in fresh, uncultured human blood using flow cytometry. It was detectable in EDTA/IL2-mac, but became obscured by the proliferating T-cells. The SRMC/NM cycle was more readily apparent in HIV-1-infected cultures, and will be further demonstrated below. Thus, a key feature of these novel cells is their ability to become NM that can, in turn, give rise to new small monocytoid cells.

### CD10 is Expressed on Developing Nurse Macrophages

**Figure 6:**
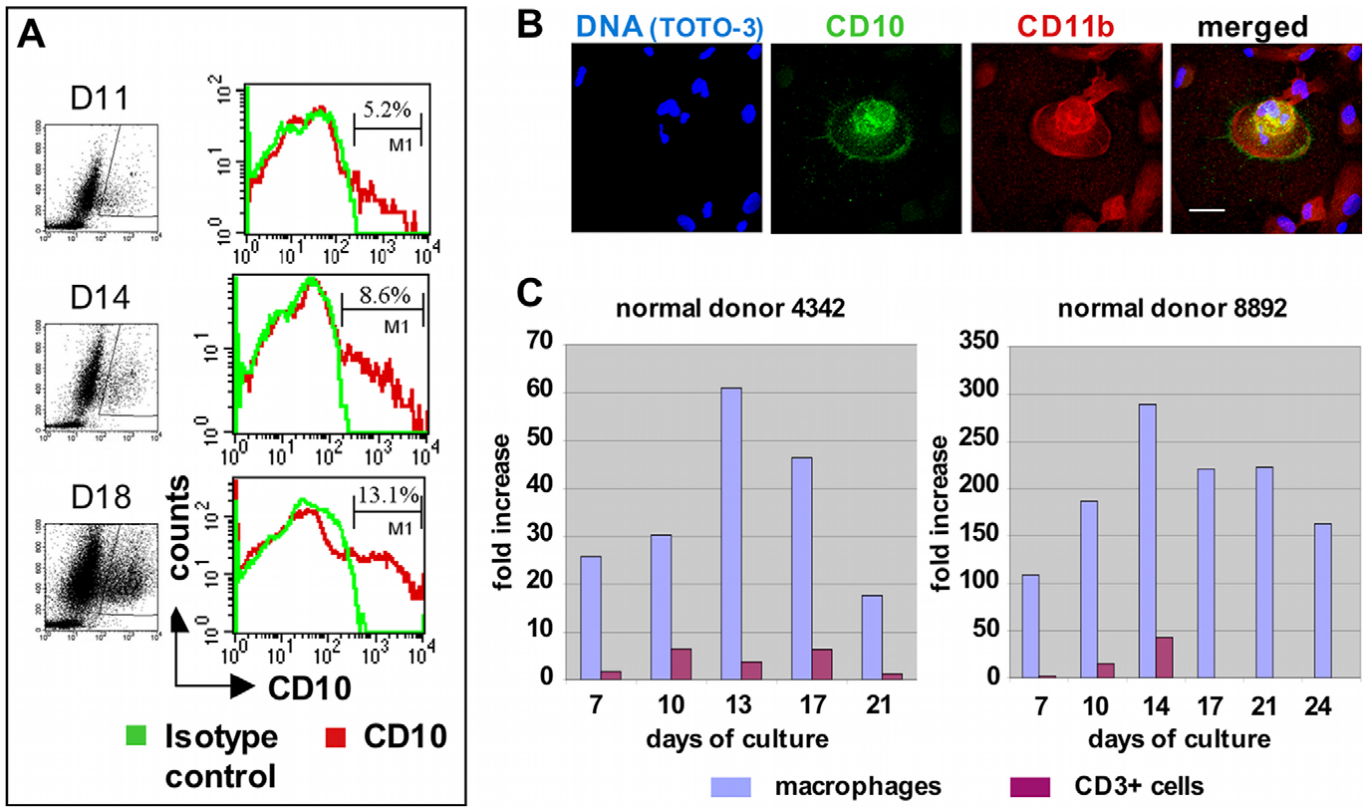
CD10 expression in cultured macrophages. (A) Flow cytometry demonstrating that SMC-derived maturing macrophages express CD10 with increasing frequency. Percentages reflect portions of maturing macrophages expressing CD10. Corresponding CD10 versus side scatter (SSC) plots with gates are shown on the left. See Table 1 for kinetics of expression. (B) Confocal microscopy images demonstrating CD10 expression on a young, EDTA-recovered nurse macrophage at D3 of IL-2 exposure. Size bar: 20 µm. (C) Expression of CD10 in macrophages and CD3+ cells recovered from primary macrophage cultures, as determined by real-time PCR. CD3+ cells were purified from the nonadherent populations using CD3+ magnetic beads. Macrophages were harvested using accutase. For each donor, the mean values obtained from uncultured CD3+ cells were subtracted from the mean values shown for CD3+ cells at each time point, and similarly, the mean values obtained from uncultured CD14+ cells were subtracted from the mean values obtained for the cultured macrophages at each time point. No values are shown for CD3+ cells for donor 8892 at days 17, 21 and 24, because too few nonadherent cells were recovered to permit CD3+ magnetic bead selection. The average percent difference between replicate reactions (including those for GAPDH) was 0.6% (donor 4342) and 0.7% (donor 8892).

Greater than 80% of nonadherent cells in primary MDM cultures from day 11 onward were highly viable maturing macrophages. Their average size, as determined by forward scatter, increased with time. Also, an increasing percentage of these expressed CD10 (Fig. 6A and Table 1). Moreover, confocal microscopy revealed CD10 expression on adherent multinucleated cells with the morphology and features of NM (Fig. 6B *)*. Furthermore, real-time PCR demonstrated CD10 expression in cultured macrophages with kinetics consistent with those observed using flow cytometry (Fig. 6C). CD10 is normally expressed on thymic epithelial cells and bone marrow stroma, but not macrophages, and it promotes early T-cell development [44], [45]. CD10 also characterizes early B-cells and late-stage neutrophils, but maturing macrophages lacked other markers of these lineages. (Data not shown.) Thus, we infer that CD10 expression identifies macrophages transitioning or committed to nurse cell behavior.

### DNA Synthesis in Nurse Macrophages

**Figure 7:**
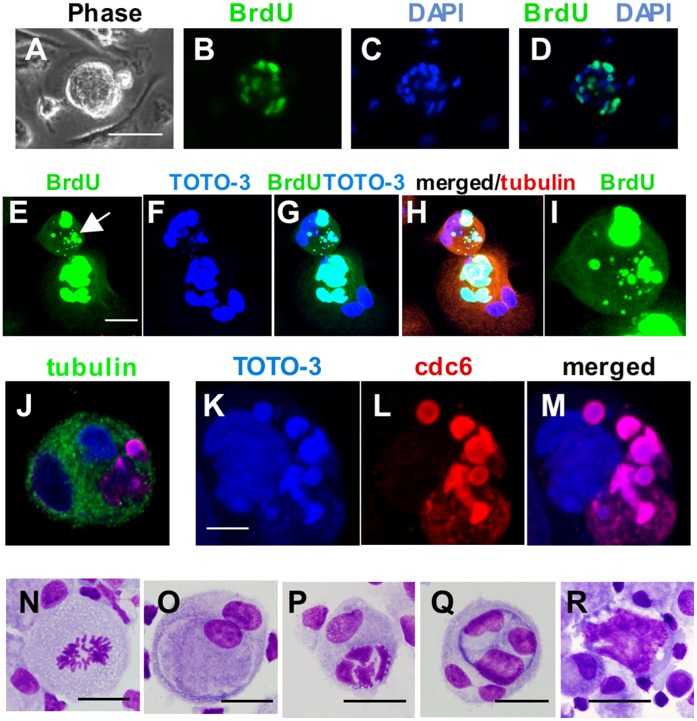
DNA synthesis and compartmentalization within multinucleated macrophages. (A-D) DNA synthesis, as evidenced by BrdU labeling, is occurring in multiple nuclei within a macrophage with nurse cell morphology. The phase contrast view (panel A) illustrates the nurse-like morphology of the living cell, prior to fixation. The culture was exposed to BrdU for 48 hours. (E-I) Confocal microscopy images demonstrating BrdU labeling in multinucleated macrophages. Culture was exposed to BrdU for 48 hours. Note that quiescent nuclei (blue only) are also present within both cells actively replicating DNA. Also apparent in the upper, somewhat smaller cell, are BrdU-labeled globular structures of multiple sizes. These colocalize with the DNA staining, confirming that they are active sites of DNA replication and not artifact. Panel I shows an enlarged view of this cell. (J-M) Confocal microscopy images demonstrating colocalization of expression of cell division cycle 6 (cdc6) protein with globular structures of DNA. Panels K-M are enlargements of the cell shown in Panel J. A gallery view of this cell is presented in Figure S5. (N-R) DNA configurations and compartmentalization in Wright’s- Giemsa stained cultured macrophages. Size bars: (A) 50 µm, (E) 20 µm, (K) 10 µm and (N) through (R) 25 µm.

The small cells enclosed within and budded from NM could arise *de novo* or derive from cells engulfed. *De novo* generation would require DNA synthesis. To detect synthesis, we performed bromodeoxyuridine (BrdU) labeling experiments and immunostaining for associated proteins. Multiple BrdU-labeled nuclei enclosed within NM were observed (Fig. 7A–D). Along with BrdU-labeled nuclei, variable-sized BrdU-labeled globular structures of DNA were also observed (Fig. 7E–I *).* An enlarged view of these structures is shown in Fig. 7I. Immunostaining demonstrated colocalization of cell division cycle 6 (cdc6) expression with globular DNA (Fig. 7J–M), further confirming that these globular structures were sites of DNA synthesis (see Figure S7 for enlargements and galley view of the entire cell). Morphological evaluation of cells harvested on sequential days, and stained with Wright’s-Giemsa, indicated that mitosis in the absence of cytokinesis initiated NM development (Fig. 7N), and was followed by formation of two distinct compartments, each containing one nucleus (Fig. 7O). Subsequently, DNA replication proceeded in one compartment, while the other remained quiescent (Fig. 7P–Q and G-H). Macrophages filled with large quantities of DNA that appeared as a single mass became apparent (Fig. 7R). Mitosis was not a feature of this replication, suggesting a mechanism leading to NM polyploidy distinct from the endomitosis that characterizes megakaryocytes.

### SRMC are Susceptible to HIV Entry and Permissive for HIV Replication

**Figure 8:**
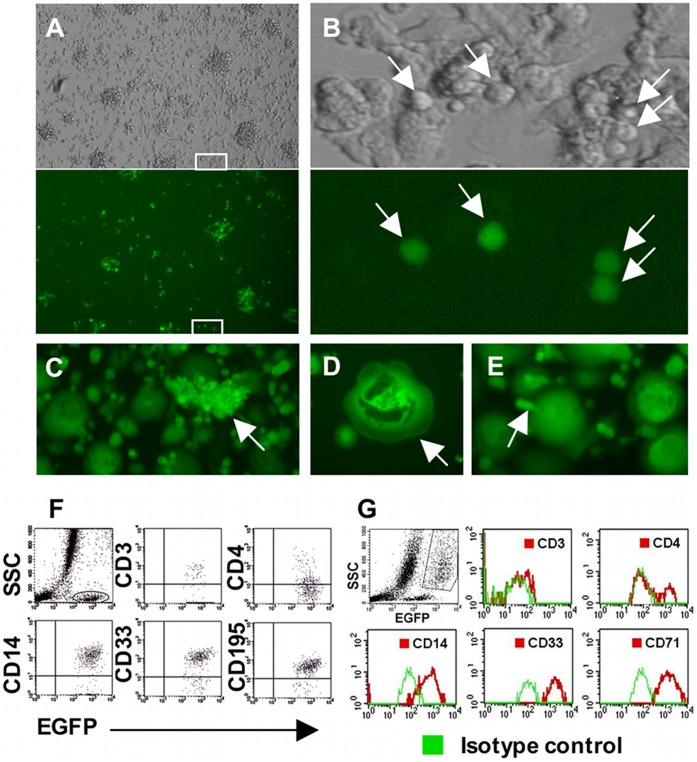
Small monocytoid cells are susceptible to HIV-1 entry leading to productive infection. (A, B) EGFP expression, a marker of productive HIV-1 infection, in cultured MDM at day 5 post infection. Upper panels photographed using phase contrast, and lower panels, which depict identical fields, using fluorescence microscopy. Areas boxed in white in (A) correspond to enlargements shown in (B). The virus-expressing cells (arrows in B) are very small adherent cells at this time. (C) Cluster of tightly associated virus-expressing small cells (arrow) at day 12 post infection. (D) Virus-expressing large macrophage with nurse cell morphology (arrow) at day 21 post infection. (E) Small, HIV-1-expressing cell budding from an infected large macrophage (arrow). (F) EGFP expression in nonadherent small monocytoid cells harvested at day 23 post infection. Small EGFP+ cells were gated as shown in the upper left panel. The other plots were based on the gated population. As can be seen, these small cells express CD14 and CD33 and lack CD3, indicative of a macrophage lineage phenotype. In the upper center plot, most of the EGFP+CD3- cells hug the X-axis. (G) EGFP expression in maturing macrophages harvested at day 9 post infection. The gated population of large macrophages is identified in the upper left panel. Because large macrophages have high levels of autofluorescence, the data are shown with isotype controls. Magnification: (A) x100, (C) x200, (D, E) x400.

Previously, using immunostaining for HIV-1 p24 antigen in cultures of MDM infected with several macrophage-tropic isolates, we observed that only a fraction of macrophages are susceptible to productive HIV-1 infection. In experiments described here, we used a macrophage-tropic HIV-1 strain (HIV-1 pSF162R3) harboring the enhanced green fluorescent protein (EGFP) gene [46]. HIV-1 expression, as evidenced by EGFP expression, was first detectable in primary MDM cultures approximately 48 hours post exposure to HIV-1 pSF162R3, and was present almost exclusively in very small adherent cells (Fig. 8A and B). Subsequently, large clusters of these small cells developed (Fig. 8C). The virus-expressing small cells, including those that clustered, developed into mature macrophages and multinucleated giant cells (MNGC), while continuing to express HIV-1 (Fig. 8C and D). Moreover, virus-expressing small cells budded from infected MNGC and then became adherent (Fig. 8E). These properties suggested that the small cells were SRMC. This possibility was confirmed by immunophenotyping performed on nonadherent cells recovered from infected cultures. Most EGFP-expressing small cells exhibited the immunophenotype of SRMC, including surface expression of CD14, CD33 and CD195, but not CD3 (Fig. 8F). Downregulation of CD4 expression in this population was also observed, as is typical in HIV-1-infected cells [47]. A small number of CD3+ EGFP+ cells are also apparent in Fig. 8F. Their origin and mode of infection is unclear, especially since they were harvested from a 30-day-old culture. They could (1) derive from an HIV-1-infected T-cell-producing NM, (2) represent persisting or NM-derived T-cells that became infected via virus-expressing macrophages some days following virus exposure or (3) typify residual T-cells that become infected during initial virus exposure, and somehow survived in the presence of virus expression. No exogenous IL-2 was added to these cultures, so whether these virus-expressing T-cells were in cell cycle remains in question. HIV-1 expression in the maturing macrophage population, with loss of CD4, was also confirmed (Fig. 8G). Small EGFP-expressing adherent cells persisted in the cultures for at least 4 weeks. Frequent observation of marked areas of the culture flasks clearly showed that the EGFP-expressing small adherent cells increased in size and differentiated into macrophages. Additionally, serial transfer of nonadherent cells from infected cultures to fresh flasks resulted in establishment of further generations of macrophages producing small monocytoid cells, although the longevity of this phenomenon was more limited, in comparison to that observed in uninfected cultures.

**Figure 9:**
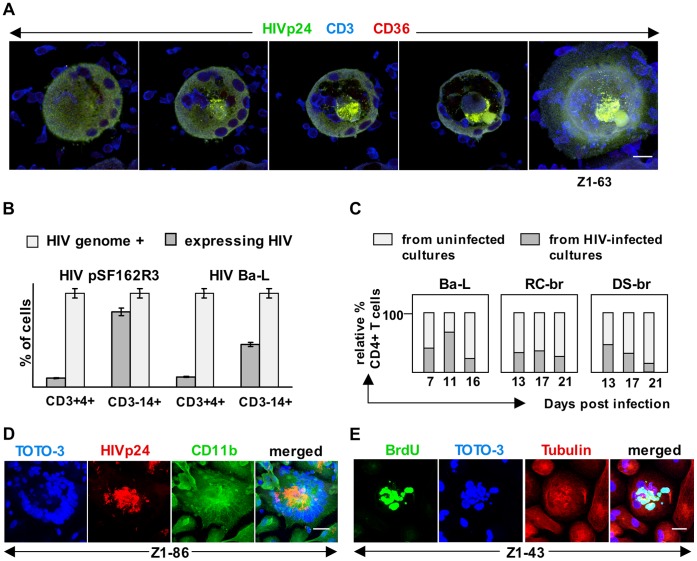
HIV-1 infection dysregulates T-cell development in nurse macrophages. (A) Confocal microscopy images showing an HIV-1-expressing small monocytoid cell (CD36+) budding from the center of a virus-expressing nurse macrophage, as evidenced by colocalization of HIV-1p24 with CD36. Also apparent is that the developing and post-budded CD3+ cells are not expressing HIV-1. This MDM culture was infected with HIV-1 5E14BM, an isolate from bone marrow, and photographed on ∼day 21 post infection. Size bar: 20 µm (B) Latently infected CD4+ T-cells are released from nurse macrophages. Numbers of genome-positive cells were determined by real-time PCR using ACH-2 cells as standards, and then normalized to 100%. Numbers of HIV-1-expressing cells were determined by flow cytometry. The numbers of CD14+ cells harboring HIV genomes also exceeded the numbers expressing HIV-1, but the relative difference was less than that seen for CD4+ T-cells. (C) Fewer CD4+ T-cells are released from nurse macrophages in MDM cultures infected with HIV-1. Nonadherent cells were harvested from infected and uninfected cultures, counted and immunophenotyped using flow cytometry. Cells from 3 or more replicate flasks were pooled. For each harvest, CD4+ T-cell numbers from uninfected flasks were normalized to represent 100%, and the numbers recovered from infected flasks were normalized, proportionately, to represent percent relative to uninfected control. (D) Confocal microscopy illustrating an HIV-1p24-expressing multinucleated giant cell in an MDM culture at D6 following infection with HIV-1 Ba-L. This cell has more than 65 nuclei. The numbers of these cells increased with time. Size bar: 20 µm (E) Abundant BrdU labeling in an HIV-1 Ba-L-infected giant cell. This labeling colocalizes with DNA. Cells were exposed to BrdU for 48 hours, beginning at day 12 post infection. 20 µm. Z numbers indicate the composite Z-stack levels for the images.

The release of HIV-1-expressing small monocytoid cells from infected NM was also observed using confocal microscopy (Fig 9A.) (We consider these large multinucleated macrophages to be NM because they enclosed CD3+ cells. The relationship between NM and HIV-1+ MNGC will be addressed below.) Typically, a single small monocytoid cell budded from the NM, often from the center. These central areas also showed the greatest abundance of HIV-1p24. We further observed CD3+ cells enclosed within, and budding from, NM (Fig. 9A; gallery view in Fig. S8). Moreover, we observed individual HIV-1-expressing NM simultaneously producing both virus-expressing SRMC and non-expressing CD3+ cells, suggesting that NM might be a source of latently-infected CD4T (Fig. 9A, Fig. S8). To further pursue this possibility, real-time PCR was used to quantitate HIV-1 DNA copy number in CD3+ and CD3- cells separated from nonadherent cell populations recovered from HIV-1-infected MDM cultures. The number of viral genome-positive cells was compared to the number of virus-expressing cells. Genome-harboring CD3+ CD4+ cells exceeded the number expressing HIV-1, typically by approximately an order of magnitude (Fig. 9B). The CD3- CD14+ population also contained latently infected cells, but in lower abundance.

### Impact of HIV-1 Infection on Nurse Macrophage Production of T-cells

To investigate the impact of HIV-1 infection on CD4T production in NM, we examined nonadherent cells harvested over time from infected and uninfected primary MDM cultures. Infection with each of three different macrophage-tropic strains led to decreased numbers of CD4T (Fig. 9C). This was accompanied by the appearance of HIV-1-expressing MNGC, whose numbers increased with time (Fig. 9D). Abundant DNA synthesis was also detected within MNGC (Fig. 9E), including in association with globular structures of varying sizes, as was observed in uninfected NM.

**Figure 10:**
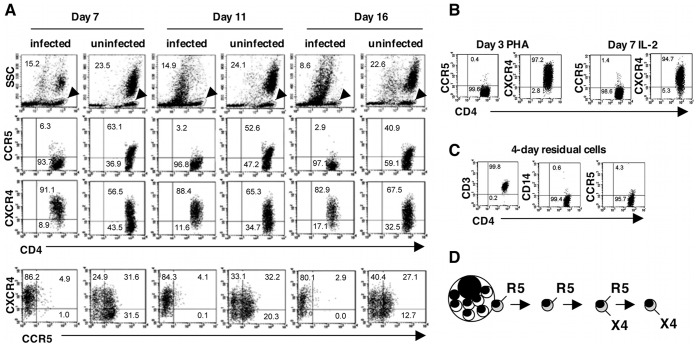
CCR5 and CXCR4 expression on CD4+ T-cells in HIV-1 infected and uninfected MDM cultures. (A) HIV-1 infection leads to preferential loss of CCR5+ CD4+ T-cells. Populations shown were gated based on low side scatter and CD4 expression. The circumscribed gated cells, identified by arrowheads, are shown in the upper left panels for each time point. MDM cultures were infected with HIV-1 Ba-L and the nonadherent cells harvested for analysis using flow cytometry. (B) The CD4+ T-cells present in PHA-stimulated cultures of normal donor PBMC are CCR5 negative and CXCR4+. This is also the case for CD4+ T-cells stimulated with PHA for 3 days, and then cultured in the presence of IL-2 for an additional 7 days. (C) CCR5 expression is also lacking on residual CD4+ T-cells harvested from uninfected macrophage cultures established from seeded PBMC. (D) Scheme for developmental stages of CCR5 and CXCR4 expression on nurse macrophage-derived CD4+ T-cells.

As noted earlier, the progressive CD4T decline that characterizes HIV-1 and SIV infection appears primarily attributable to loss of CCR5+ CD4T. We examined the impact of HIV-1 infection on production of CCR5+ and CXCR4+ CD4T in MDM cultures (Fig. 10A). In uninfected cultures, both CCR5+ and CCR5+ CXCR4+ (dual positive) CD4T were highly represented, together accounting for the majority of CD4T present throughout the culture period. In striking contrast, at all times surveyed post infection, almost no CD4T expressing only CCR5 were detected in infected cultures, and the number expressing both CCR5 and CXCR4 was significantly reduced. In other experiments, and consistent with published reports [48], we detected only CXCR4+ CD4T in both PHA-stimulated and IL-2 maintained PBMC cultures (Fig. 10B); this was similarly true for residual T-cells recovered from initial seedings of PBMC for macrophage cultures (Fig. 10C). Together, these findings suggest that CCR5+ CD4T recovered from MDM cultures derive directly from NM. From these and other data, we deduced a maturation scheme for expression of these chemokine receptors on NM-derived CD4T (Fig. 10D). This is discussed below.

## Discussion

**Figure 11:**
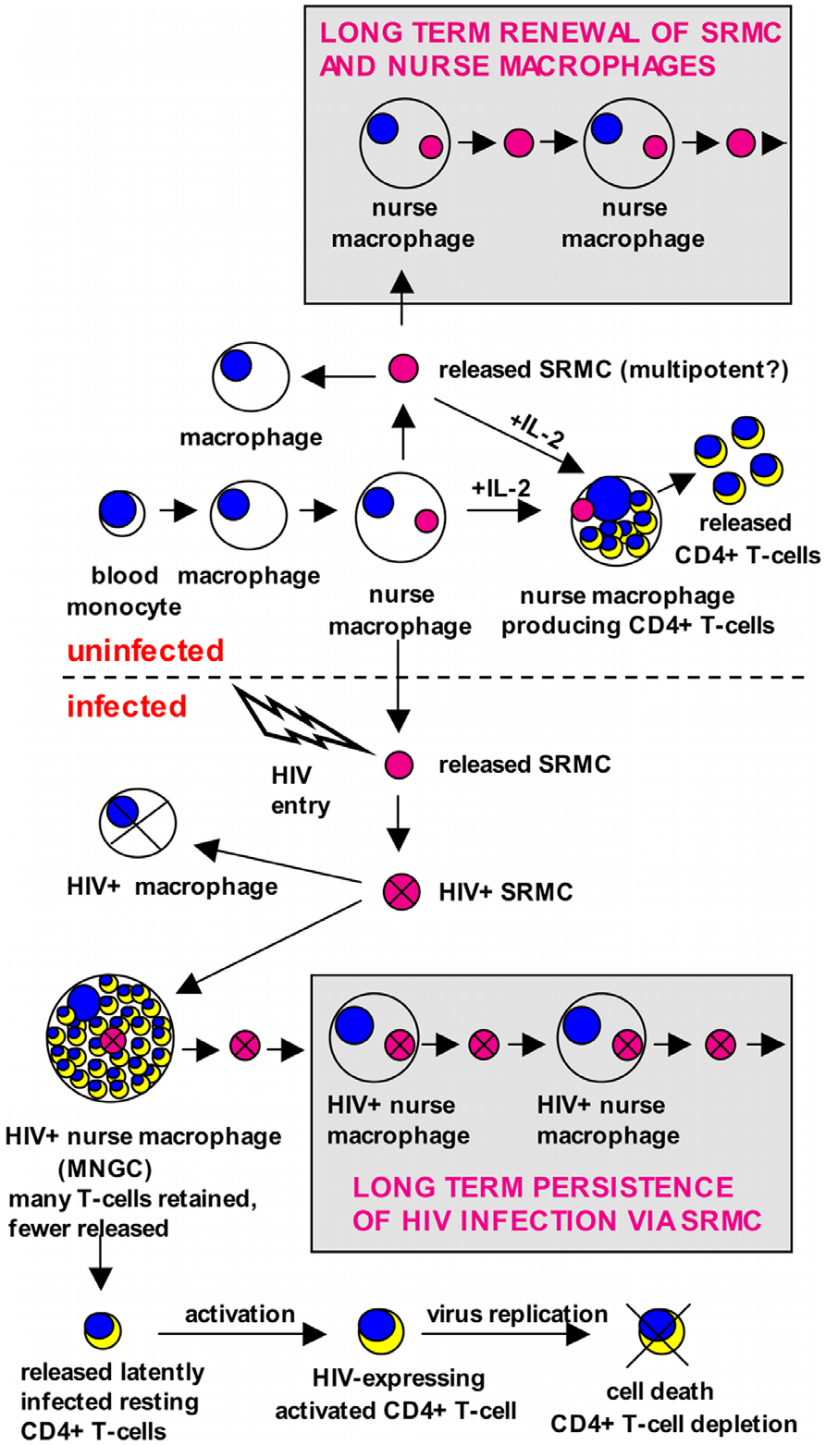
A model for nurse macrophage capabilities and role in HIV-1 pathogenesis. *In vitro,* we observed that human blood monocytes differentiate into macrophages, and that a portion of these can further develop into what we term “nurse macrophages.” These nurse cells can generate within themselves, CD4+ T-lymphocytes, as well as a previously unknown small monocytoid cell (SRMC). The SRMC, itself, can develop into a nurse macrophage with the ability to give rise to another SRMC. The NM/SRMC cycle, then, represents a form of self-renewal. When cultures of human macrophages are exposed to HIV-1, the SRMC become productively infected, but virus expression does not kill them. Rather, they can differentiate into macrophages, including nurse macrophages, in the presence of virus expression. Thus, a NM/SRMC cycle is established that is characterized by ongoing HIV-1 expression. In the absence of HIV-1 infection, nurse macrophages release resting CD4+ T-cells. The development and release of these lymphocytes is enhanced by treatment with IL-2. When productively infected with HIV-1, the nurse macrophage retains, rather than releases the T-lymphocytes and as a consequence, it takes on the appearance of a multinucleated giant cell. We propose that these events may recapitulate events that occur *in vivo* and if so, this model offers explanations for HIV-1 persistence and latency.

We present a model for nurse macrophage development and capabilities, and for how HIV-1 utilizes and impacts this process (Fig.11). Key features of this model include (1) development of a NM from a blood monocyte, (2) *de novo* generation within the NM of a novel small monocytoid cell (SRMC) that is subsequently released, (3) generation and release of CD4T from a NM, (4) maintenance of a cycle of NM/SRMC production, which serves as a form of self-renewal, (5) HIV-1 entry into SRMC which leads to productive infection and establishment of a NM/SRMC cycle that maintains viral persistence, (6) dysregulation of CD4T production in HIV-1-infected NM, the consequence being retention rather than release of CD4T and (7) the release of latently infected CD4T that can become targets for cellular activation, followed by HIV-1 expression and death. Thus, HIV-1 infection of SRMC sets the stage for both establishment of long-term viral persistence and depletion of CD4T. As noted earlier, the ability of macrophages to produce new cells is not without precedent. Avian osteoclast-like multinucleated cells, derived from blood monocytes, can give rise to mononuclear cells via budding [9]. Importantly, this avian system exhibits two key novel features analogous to those we observed in human NM, (1) the ability of the newly generated mononuclear cells to give rise, by themselves, to a second generation of multinucleated giant cells and (2) the fact that this generation occurs in the absence of traditional mitosis.

Does the phenomenon of T-cell production by NM mimic events that take place in thymus during either fetal development or adulthood, or does it reflect a process that occurs more generally, in lymphoid and perhaps even nonlymphoid tissues? Whether the molecules and steps involved in T-cell development gleaned from murine studies are applicable to NM behavior remains to be determined. Generation of the T-cell repertoire via positive and negative selection is thought confined to the thymus, with the specialized thymic epithelial cells playing key roles. Establishment of the repertoire takes place mostly during fetal development and early life, with thymic involution in adulthood. Genetic recombination of germline DNA to yield functional T-cell receptors is necessary for the generation of competent T-cells. The VDJ recombination events occur randomly and it has been estimated that 98–99% of all thymocytes fail to survive selection and die before exiting the thymus. Pre-TCRα is present in immature, but not mature T-cells, reflecting rearrangement of the TCR β-chain prior to that of the α-chain. Our detection of pre-TCRα expression within the macrophage-enclosed developing T-cells, as well as detection of RAG1 expression and TREC in macrophages, suggests that TCR rearrangement was taking place therein. Interestingly, pre-TCRα mRNA, along with CD3 mRNA, has been detected in murine thymic dendritic cells, leading to the suggestion that these cells have shared a developmental past with T-lymphocytes [49]. Moreover, there is evidence that VDJ rearrangement first begins in uncommitted hematopoietic progenitors, prior to T-cell/B-cell/NK cell/dendritic cell divergence [50], [51]. We also detected CD2+CD3+CD16+CD32+ cells with increasing frequency during the macrophage culture period. This population was not present in PBMC and given that CD16 and CD32 are molecules normally expressed on macrophages, but not T-cells, their presence on T-cells could reflect molecules carried along as the T-cells exit from NM. Indeed, we found that CD16 and CD32, but not CD2 and CD3, were lost from the surface of these quadruple-positive cells, following sorting and culture in IL-2. (As noted before, CD16 and CD32 are expressed on early double negative fetal mouse thymocytes [33], [34].) Additionally, the observed sequestration of quiescent and synthetically active DNA into distinct compartments within NM could permit TCR rearrangement to occur in one compartment, while germline DNA is preserved in the other. Although the idea that T-cell receptor rearrangement events can occur within a macrophage might seem outlandish, this possibility is reminiscent of an intriguing behavior of *Tetrahymena thermophila*, which has both a micronucleus and a macronucleus. The micronucleus possesses the germline DNA and the macronucleus, which derives from the micronucleus via endoreplication, contains 23 times more DNA and a sequence complexity reduced by 10–20% [52]–[54]. Most important and relevant, corresponding regions of the micro- and macronucleus have been cloned and sequenced and revealed that the macronucleus derives from internal deletions that occur within the micronucleus genome [52], [55]. This deletion of specific germline sequences is analogous to the process that characterizes the TCR and immunoglobulin gene rearrangements. To further extend this, we speculate that not only does the compartmentalization of DNA observed in NM set the stage for confinement of genetic rearrangements to one compartment, but that the small SRMC, like the *Tetrahymena* micronucleus, is tasked with preserving germline DNA. Our findings raise another intriguing question. Can the presence of antigen within a NM drive VDJ rearrangement such that T-cells develop within which carry TCR with specificity for that antigen? If so, then a macrophage could, in essence, by producing T-cells of appropriate specificity, instigate an immune response without having to depend on making contact with a specific passer-by T-cell. This possibility represents a seemingly efficient mechanism.

Maintenance of T-cell production in the thymus requires replenishment with a bone marrow-derived population that enters via blood. The exact nature and characteristics of these entering progenitor T-cells remain elusive. Evidence suggests that they enter as multipotent cells, and commit to the T-cell lineage only after entry into the thymus [56]. A CD4low population with germline TCR genes has been isolated from adult murine thymus [57]. This population was scarce, representing only 0.05% of all thymocytes, and it provided for extensive reconstitution of both αβ and γδ lineages following intrathymic transplantation. Only T-cells were produced in the irradiated thymus, but this CD4low cell had the ability to develop into T and B-cells, but not myeloid cells, in recipient animal bone marrow, spleen and lymph node, following intravenous injection [58]. Subsequently, it was shown that these CD4low T precursors express CD16 (FcγIII) and CD32 (FcγII) and that this expression defines an early DN stage that precedes Vβ(Dβ)Jβ rearrangement [33], [34]. Although it is not clear if T-cell development in human thymus parallels that in mice, SRMC exhibit a CD4lowCD16+CD32+ phenotype and would likely be sparsely represented within thymus, where they could develop from NM that enter as blood-derived monocytes. Given that SRMC do not express CD3 and can mature into macrophages that give rise to another generation of SRMC, it is likely that they contain germline DNA, as do the early CD16+CD32+ murine thymic precursors. SRMC could develop into T-cell-producing NM within the thymus and thus, in essence, represent a T-cell precursor in that location. The fact that SRMC appear undetectable in blood may help to explain the difficulty in identifying the T-cell precursors that circulate. Of potential relevance, a human CD34+CD10+CD24- cell with the potential to develop into lymphoid cells, has been detected in bone marrow, cord blood, thymus and blood [59]. Cells with a dendritic morphology could develop from this population [59]. We have detected CD34+ cells in low abundance in our macrophage cultures (Fig. S3), but it is not yet clear if these cells also express CD10, as do NM. Thus, while our data indicate that the CD4+ T-cells in our macrophage cultures arose from NM, we acknowledge the possibility that our observations might be attributable to an as yet unrecognized circulating cell of low abundance with the ability to serve as a T-cell precursor. Also, we recognize the value in using the mouse system to investigate our hypotheses regarding T-cell development, especially given the wealth of knockout animals and other reagents available.

Although the thymic nurse cell concept precipitated our initial experiments, the relationship between thymic nurse cells and NM is unclear, as is the relationship between thymic nurse cells and thymic epithelial cells, albeit both express keratin and MHC-II. As noted earlier, although Wekerle and Ketelsen found that thymic nurse cells and macrophages had identical patterns of surface antigen expression, they concluded that these cells were not macrophages because they lacked phagocytic activity and behaved more like epithelial cells [2], [4]. We observed that NM could assume an epithelioid appearance in response to IL-2, exhibit reduced phagocytic activity, and express CD10, a molecule found on thymic epithelium, raising the possibility that thymic nurse cells and nurse macrophages are similar entities. Studies of murine fetal thymus have revealed that medullary epithelium is generated during ontogeny from epithelial stem/progenitor cells, that each progenitor generates one medullary “islet” and that very early T progenitor cells are sufficient for islet development [60]. It has also been demonstrated that a single embryonic epithelial cell can give rise to both cortical thymic epithelial cells and medullary thymic epithelial cells [61], and current views holds that thymic epithelium derives from a single endodermal germ layer [62]. The phenotype of thymic epithelial progenitors, however, is unclear, as is their persistence beyond fetal development. We postulate that SRMC may be a source of thymic epithelial progenitors. They were more responsive to treatment with IL-2 than the replated large multinucleated macrophages, sending out more processes more quickly, and they could develop into T-cell-producing NM. This possibility could help to explain how thymic epithelial cells “educate” developing T-cells-they do so by serving as sites for the actual generation of the T-cells. Interleukin-2 is produced only by T-cells, so thymus represents the richest microenvironment for this induction. Thus, thymus represents an obvious candidate site where NM production of CD4T might occur. Conceivably, the process in this location could occur during fetal development, and then persist, at some level, during adulthood. We also propose that mucosal tissue and gut-associated lymphoid tissue are possible candidate sites. This supposition is supported by the fact that these tissues are rich sources of CCR5+ CD4T. (The T-cells produced *in vitro* by NM are CCR5+ CD4T. Few circulating CD4T are CCR5+.).

Several studies have suggested that blood monocytes are pluripotent or can behave as “progenitor” cells with the capacity to differentiate into hepatocytes, pancreatic islet cells and neuronal cells, as well as cells expressing markers of bone, muscle, cartilage, fat and endothelium [63]–[68]. The presence and production of SRMC in macrophage cultures can readily escape notice, raising the possibility that the pluripotency and progenitor behavior ascribed to monocytes may actually be attributable to SRMC. Monocytes are known to enter essentially all tissues, where they differentiate into macrophages, in some cases, specialized macrophages such as the Kupffer cells found in liver. The tissue microenvironment likely plays a role in the induction of macrophage specialization. It is conceivable that the fate or pathway of terminal differentiation of an SRMC produced and released by a NM might be determined by the microenvironment in which it finds itself. That is, if the lineage plasticity of monocyte/macrophages observed *in vitro* is operational *in vivo*, and if that plasticity is actually attributable to SRMC, at least in part, then an SRMC produced from a NM in the liver might develop into an hepatocyte, or hepatocyte progenitor. In such a situation, the number of rounds of the NM/SRMC cycle might be limited, since lineage committed hepatocytes/hepatocyte progenitors could continue the production of cells alone. Obviously, *in vivo* experiments are required to determine the physiological relevance of the phenomena we have observed *in vitro*. A key question is “Do SRMC exist within the human body?” Of possible relevance to SRMC are the very small embryonic-like (VSEL) stem cells, first described in mice by Kucia and colleagues [69]. These cells have also been found in human cord blood [70], and they appear to be mobilized to blood in patients experiencing myocardial infarction [71] and stroke [72].

We propose that SRMC can “renew” themselves via the NM/SRMC cycle. Does this mode of self-renewal have relevance to hematopoietic stem cell renewal? Interestingly, we detected small numbers of CD34+ cells among nonadherent populations recovered from 4–6-week old primary macrophage cultures reseeded at low density (Fig. S3). (They represented 0.25% of the total nonadherent population.) Maintaining human CD34+ cells *in vitro* for extended periods of time, particularly in the absence of any hematopoietic growth factors and without differentiation, is challenging. This raises the question of whether the CD34+ cells we detected arose from NM, a possibility under active investigation in our laboratory. Even in bone marrow, their primary residence, CD34+ cells are very limited in number, typically representing 1–2% of total cells, but yet, current understanding suggests that this population provides for lifelong production of red and white blood cells. We reason that if NM could produce CD34+ cells, this mechanism would represent an efficient and responsive way to maintain indefinite renewal of hematopoietic progenitors without those progenitors having to either divide, or remain quiescent for long periods of time. We are currently attempting to developing quantitative methods, including clonal methods, to determine the longevity of the NM/SRMC cycle, and if NM can generate CD34+ cells. Important to note is that we do not believe that SRMC directly develop into monocytes, but that monocytes arise from the well-known pathway that originates with CD34+ cells. Still unclear to us, however, is whether all, or only some, marrow-derived circulating monocytes have the ability to develop into NM.

Macrophages are thought to be terminally differentiated cells with limited potential for DNA replication. Our BrdU labeling and cdc6 immunostaining experiments demonstrated DNA synthesis in NM, and further revealed synthesis associated with variable-sized globular structures of DNA, and compartmentalized sequestration of quiescent and replicating DNA. These unusual structures, the scarcity of mitoses, the presence of giant macrophages filled with DNA that appeared as a single mass, and the rate at which new cells were produced, suggests a process of DNA replication unlike that which typically occurs in proliferating cells. Although not yet documented in human cells, endoreplication seems a reasonable candidate mechanism for this DNA synthesis, especially since it shares the characteristics we observed in NM and is a process that leads to polyploidy [73]. Experiments are now underway in our laboratory to investigate this possibility.

Using an infectious macrophage-tropic HIV-1 reporter virus, we found that SRMC, but not more differentiated monocyte/macrophages, are susceptible to HIV-1 entry that results in productive infection with detectable virus expression approximately 48 hours post exposure. Two conditions provide for highly efficient infection of SRMC–-their surface expression of CCR5 along with levels of CD4 that exceed those on blood monocytes, and the fact that they are in cell cycle as they exit from NM, and shortly thereafter, as evidenced by expression of cdc6 and other indicators. This later condition can provide for integration of the HIV-1 genome. We have observed that most circulating monocytes are CXCR4+ and lack CCR5. Their lack of susceptibility to productive infection with CXCR4-utilizing HIV-1 strains further emphasizes the importance of when, relative to CCR5 or CXCR4 expression, the monocytoid cell is cycling. We also observed that HIV-1-expressing NM can give rise to virus-expressing SRMC and that these SRMC can, in turn, develop into virus-expressing NM capable of producing another generation of infected SRMC. Perpetual cycles of NM/SRMC production *in vivo* could maintain HIV-1 within the body indefinitely. Importantly, this mode of persistence would not require new rounds of direct infection and thus, would be poorly amenable to the effects of most antiretrovirals. In the natural history setting, HIV-1 infection is typically characterized by a chronic phrase, in which there is slow loss of CD4T. Our model is consistent with this time course. We propose that during the acute phase of infection, a percentage of SRMC become infected, and thereby establish an indefinite infection via the NM/SRMC cycle. Over time, as “called upon” to produce CD4T through an as yet unidentified stimulus, the infected NM/SRMC fail in their mission. The process of CD4T development and/or release is interrupted. With time, this leads to a cumulative loss of CD4T. In addition, CD4T released from infected NM could harbor HIV-1 genomes, as our data indicate. Such cells would be susceptible to virus-mediating killing, should they become activated and start to express the virus. Clearly, patient studies are required to substantiate the relevance of our model.

Naive and memory CD4T are frequently identified by expression of CD45RA or CD45R0, respectively. CCR5 and CXCR4 expression on T-cells is largely reciprocal, CCR5 being associated with the memory phenotype, and CXCR4 with naïve cells [48]. HIV-1 infection is characterized by the presence of a pool of latently infected resting CD4T harboring integrated replication-competent viral genomes [13], [14], [74]. Because these are predominantly memory cells (CD45RO+), their viral latency is thought to arise from previous HIV-1 infection while activated, with subsequent return to resting. Because cellular activation would likely lead to HIV-1 replication, many of the productively infected cells would not be expected to survive. To account for this predicament, it has been postulated that some of the virus-infected activated cells, with integrated viral genomes, survive long enough to return to a resting state [74]. Evidence also suggests that direct infection of naive CD4T is not the primary mechanism leading to generation of latently infected resting T-cells [75]. We observed that CD4T exit from NM in primary MDM cultures as resting, CD45RA negative cells and moreover, we found that CD4T which develop within, and exit from, HIV-1-infected NM, can acquire viral genomes during their developmental stage, but often exit as cells not expressing the virus. Thus, these cells represent a population of latently infected resting CD4T with a memory phenotype, raising the possibility that this reservoir *in vivo* may, at least in part, derive from infected NM. Importantly, this proposed derivation circumvents the issue of survival of activated, infected CD4T with subsequent return to a resting state. Also, this mechanism links viral latency with indefinite viral persistence.

Experiments in which we examined CCR5 and CXCR4 expression on CD4T recovered from cultures of infected and uninfected primary macrophages demonstrated profound loss of CCR5+ CD4T in the presence of infection. These experiments also suggested a maturation scheme for expression of these chemokine receptors on NM-derived CD4T (Fig. 10D). Conceivably, CD4T could exit from NM bearing CCR5, CXCR4 or both molecules. However, if CXCR4+ cells exited directly, reduction in their numbers would be apparent in infected cultures, owing to virus-mediated compromise of cell release from NM. Similarly, if dual-expressing cells exited directly and subsequently lost one of the receptors, depletion of both CCR5+ and CXCR4+ cells would be apparent. The most likely scenario, then, is that CD4T bud from NM as CCR5+ cells. Their lack of CD71 expression, nonproliferative status and demonstrated release as latently infected cells, indicates they are resting cells at this stage and thus, not susceptible to productive HIV-1 infection. The released CCR5+ CD4T then acquire CXCR4 to become dual positive. Subsequently, CCR5 is lost. This profile mirrors that observed when NM are treated with IL-2, as well as when T-cells are activated *in vitro* (Fig. 9B). These activated T-cells are CXCR4+ and lack CCR5. This relationship between activation and stage of chemokine receptor expression indicates that the primary cause of CD4T depletion in this *in vitro* system is not infection post exit, but failure to exit from NM. Classic, HIV-1-associated MNGC formation is the consequence.

Multinucleation characterizes both HIV-1-infected MNGC and NM, but normal, uninfected NM contain many fewer nuclei. *In vitro*, HIV-1-infected MNGC often contain more than 50 nuclei, sometimes even as many as 100, in the case of highly replicative macrophage-tropic strains. HIV-1 infection of macrophages is known to result in MNGC, a process that can be driven by viral envelope-mediated cell fusion [76], [77]. However, considering the number of participating cells needed to reach the high levels of polyploidy we observed within 5–6 days of infection, along with the time required to attain the levels of virus expression necessary to mediate fusion, we propose that MNGC formation might also reflect dysregulation of cell production in infected NM. Dysregulation could represent a defect in production and/or release of cells. In BrdU-labeling experiments, we observed abundant DNA synthesis in HIV-1-infected MNGC and the morphology of the BrdU-labeled structures mirrored that seen in uninfected NM. Qualitatively, the levels appeared to exceed those in uninfected NM, but further studies are required. The nuclei in MNGC appear morphologically normal, but accumulated. This accumulation may reflect an inability of other required processes to keep pace with accelerated production of nuclei. Alternatively, infection may suppress synthesis of components essential for progeny cell assembly or release. In addition, we observed a decline in the number of CD4T produced in HIV-1-infected cultures, suggesting that fewer were released. Because these CD4T were typically not sites of active replication as evidenced by the lack of HIV-1 p24 expression, direct virus-mediated cell killing is an unlikely explanation for the decline.

Although it is not yet clear if the HIV-1/macrophage interactions we describe here mimic events *in vivo*, certain published studies support this possibility. For example, a non-opportunistic, generalized giant cell disease prevalent in SIV-infected cynomolgus macaques, has been described [78]. The giant cells expressed macrophage markers and SIV antigen, some also expressed T-cell markers, and they were most abundant in lymph node and gut mucosa, especially within the lamina propria, which is particularly rich in CCR5+ CD4T. The number, size and intracellular distribution of nuclei within these giant cells varied considerably and many nuclei were highly irregular and euchromatic, consistent with the DNA structures that we observed in NM.

This work is still in its infancy and it is not yet clear if the NM phenomenon represents a clear departure from current views of T-cell development, if it can be integrated into current models, or if it represents a previously unrecognized alternative pathway. Similarly, it is not clear if NM and SRMC exist *in vivo*. Obviously, our findings have far-reaching implications. Macrophages are located in most tissues and play key roles in numerous biological processes. They are also subject to infection with many viruses and other pathogenic microorganisms, and they have been mechanistically linked to a myriad of diseases including chronic autoimmune and inflammatory diseases. Discovery of SRMC, and recognition that macrophages can serve as sites for the *de novo* generation of other cells, can foster new understanding regarding basic aspects of macrophage biology in health and disease. Moreover, nurse macrophage biology explains many features of HIV-1 infection and disease progression. The HIV-1/NM system we describe mirrors key features of HIV/SIV infection within intestinal mucosa, making it an attractive model for the study of virus/host interactions in this tissue. Evidence now indicates that the viral set point, a marker of the rapidity of HIV-1 disease progression, is largely determined by the breadth of replication within the mucosa during acute infection and moreover, that long-term non-progression rests with restoration of mucosal CCR5+ CD4T [79]. Studies of NM infection can aid identification of mechanisms and molecules that could spark new strategies for preventing and treating HIV/AIDS.

## Materials and Methods

### Macrophage Culture

Normal donor PBMC were recovered from leukopaks using ficoll-hypaque separation. The leukopaks were obtained from the Johns Hopkins Hospital Leukapheresis Laboratory. Use of these preparations is IRB exempt. For primary macrophage cultures, these PBMC were seeded into plastic T25 flasks (Corning) at 3×10^7^ per flask, or 24-well plates (Costar) at 3×10^6^ per well, in RPMI 1640 medium supplemented with 20% fetal bovine serum (FBS) (Invitrogen), 10% pooled human serum (Cambrex/Lonza), 2 mM L-glutamine, 100 U/ml penicillin, and 100 µg/ml streptomycin. Four to 7 days later, when the macrophage monolayers were well-established, the cultures were washed multiple times to remove the nonadherent population, and then cultured further in the medium described above, but lacking human serum (RPMI-20%). Cultures were fed at 3–4 days intervals with a complete medium exchange and removal of nonadherent cells. In some cases, nonadherent cells were seeded into fresh flasks or plates to establish secondary cultures of adherent macrophages.

For EDTA/IL2-mac cultures, 4- to 6-week-old primary macrophage cultures were incubated at 37°C for 10–15 minutes with 20 mM EDTA prepared in Dulbecco’s phosphate buffered saline lacking calcium and magnesium (DPBS). Following incubation, the cells were further detached only by gentle pipetting, and FBS (20% by volume) was added, prior to centrifugation. These cells were seeded into 24-well plates at 6×10^4^–2.7×10^5^ per well in RPMI-20%. Two-three days later, the wells were washed well to remove nonadherent cells, and fed with RPMI-20%, supplemented in some wells with 20 U/ml recombinant IL-2 (Roche). Medium was completely exchanged every 2–4 days with removal of nonadherent cells. Pipetting was used to enhance removal of nonadherent cells.

Macrophage cultures were also established using CD14+ cells (monocytes) purified from PMBC using CD14 microbeads and a magnetic bead separation system (Miltenyi Biotec). The cell separations were performed according to manufacturer’s specifications. Purity of the recovered CD14+ cell populations was >95% as determined by flow cytometry. The CD14+ cells were seeded into 24-well plates at 8×10^5^ per well and cultured as described above, using RPMI-20% supplemented additionally with 10% human serum for the first 7–10 days. On day 10–14 of culture, wells were washed and IL-2 added (20 U/ml). Nonadherent cells were harvested as they became apparent, and subjected to analysis using flow cytometry. Untreated wells were maintained and examined in parallel.

Secondary macrophage cultures were used for detection of TREC. To prepare these, primary macrophage cultures of at least 4 weeks of age were incubated with accutase (Invitrogen) until most or all of the cells were detached, or could easily be detached by washing. (The length of incubation varied between 30 and 60 minutes.) The detached cells were harvested, RPMI-20% was added, and the cells pelleted by centrifugation. The washed was removed from the pelleted cells, they were resuspended in RPMI-20%, counted, and seeded into 24-well plates at a density of 3×10^5^/well. Two to three days later, the wells were washed with DPBS to remove any nonadherent cells, and IL-2 was added at a concentration of 20 U/ml. Wells lacking IL-2 were included in each experiment. The medium was changed as needed (typically every 2–4 days) with harvest of the nonadherent cells.

### HIV-1 Infection of Macrophage Cultures

For infection with HIV-1, macrophage cultures were established as described above in T25 flasks, and infected on day 7 of culture. Prior to infection, they were washed to remove nonadherent cells. To infect, all medium was removed, 1 ml of viral inoculum was added, and the flasks incubated at 37°C for 1 hour with gentle hand-rocking every 10–15 minutes to insure even distribution of the liquid. The inoculum was then removed and fresh RPMI-20% added. Infected cultures were fed as described above. The following HIV-1 isolates, propagated in primary macrophages, were used: Ba-L, recovered from post-mortem pediatric lung [10], RC-br and DS-br recovered from brain tissue [80], [81] and 5E14BM recovered in our laboratory from patient bone marrow. Also used was the EGFP reporter virus, pSF162R3, propagated in 293T cells [39]. To follow and document the kinetics of HIV-1 expression, primary cultures were exposed to pSF162R3 as described above. Beginning at 8–12 hours post virus exposure, the living cultures were examined at frequent intervals for EGFP expression using fluorescence microscopy. Cultures were photographed using a Nikon Eclipse TE2000-U microscope. In some experiments, EDTA/IL2-mac cultures were prepared from primary cultures infected with pSF162R3. In these experiments, the primary cultures were exposed to the virus on day 7 as described above, then cultured for 14–15 days prior to EDTA treatment and replating. The EDTA recovered cells were replated and cultured as described above for uninfected populations.

### Primary T-cell Culture

Normal donor PBMC were seeded into plastic T25 flasks at 2×10^6^/ml in RPMI-20% supplemented with 5 µg/ml PHA-P (Difco). Flasks were incubated in the upright position for 3 days. The cells were then centrifuged and resuspended in RPMI-20% supplemented with 20 U/ml recombinant IL-2 (Roche). Cultures were subcultured 1∶2 with a complete medium exchange at 3–4 day intervals.

### Flow Cytometry and Cell Sorting

Anti-CD135 (clone BV10A4H2) was purchased from Caltag Laboratories (now Invitrogen) and anti-CD133 (clone AC133) was purchased from Miltenyi Biotec. The following antibodies were purchased from BD Biosciences (clone numbers are shown in parentheses): anti-CD2 (S5.2), anti-CD3 (SK7), anti-CD4 (SK3), anti-CD7 (M-T701), anti-CD8 (SK1), anti-CD10 (HI10A), anti-CD11b (D12), anti-CD13 (L138), anti-CD14 (MϕP9), anti-CD16 (B73.1 and NKP15), anti-CD19 (SJ25C1, HIB19), anti-CD25 (2A3), anti-CD32 (FLI8.26), anti-CD33 (P67.6), anti-CD34 (8G12 and 581), anti-CD36 (CB38), anti-CD45 (2D1), anti-CD45RA (HI100), anti-CD45RO (UCHL1), anti-CD56 (MY31 and B159), anti-CD71 (LO1.1), anti-CD91 (A2MR-A2), anti-CD117 (104D2), anti-CD122 (TU27), anti-CD184 (12G5), anti-CD195 (2D7/CCR5), anti-HLA-DR (L243 [G46-6]), anti-TCR αβ (T10B9.1A-31) and the isotype controls IgG_1_ (40), IgG_2a_ (39) IgG_2b_ (27–35) and IgM (G155-228). Harvested cells were centrifuged, resuspended in RPMI-20%, counted and 50 µl aliquots incubated with fluorochrome-labeled antibodies for 20 minutes at RT in the dark. One ml of DPBS containing 1% FBS and 0.09% sodium azide (wash buffer) was added, tubes were briefly vortexed, and 250 µl of fixative (9.25% formaldehyde plus 3.75% methanol) was added, followed by 3 ml of wash buffer. Following centrifugation, cells were washed again, then resuspended in 1% paraformaldehyde and acquired and analyzed on a FACSCalibur cytometer using CellQuest software. Ten-to-thirty thousand events were acquired per tube, depending on the frequency of populations of interest. Analyses were carried out using the CellQuest and Paint-a-Gate programs.

Cell sorting was performed using the antibodies directed against CD2, CD3, CD16 and CD32 identified above. Nonadherent cells were harvested from primary macrophage cultures between days 14 and 22, immunostained and sorting using a FACSAria cell sorter (BD Biosciences). Analyses of the post-sorted cells was performed using FLOWJO software.

### Confocal Microscopy

The primary mouse monoclonal antibodies used were: anti-CD3 (clone PS1, IgG2a, Novocastra, Ltd, distributed by Vector Laboratories), anti-CD10 (clone HI10A, IgG_1_, BD Biosciences), anti-CD11b (clone 238446, IgG_2b_, R & D Systems), anti-CD36 (185-1G2, IgG_2a_, Santa Cruz Biotechnology), anti-CD68 (KP1, IgG_1_ Novocastra), anti-MHC II (anti-human HLA-DP/DQ/DR clone CR3/43, IgG_1_, DAKO), anti-HIV-1p24 (Kal-1, IgG_1_, DAKO), anti-α-tubulin (236-10501, IgG1, Molecular Probes) and anti-cdc6 (37F4, IgG2a, Molecular Probes). The two polyclonal antibodies used were: anti-pre-TCRα (C-17, goat polyclonal IgG, Santa Cruz Biotechnology) and anti-CD3 (rabbit polyclonal, Novocastra). Subclass specific secondary antibodies were used in combination with the primary monoclonal antibodies. The following secondary antibodies were purchased from Jackson ImmunoResearch Laboratories, Inc.: Rhodamine Red-X (RRX)-conjugated affinity purified goat anti-mouse IgG subclass 1 specific (used with anti-CD68 and anti-MHC II), Cy2-conjugated affinity purified goat anti-mouse IgG subclass 2a specific (used with the mouse monoclonal anti-CD3), Cy3-conjugated affinity purified goat anti-mouse IgG subclass 2a specific (used with anti-CD36 and anti-cdc6), Cy5-conjugated affinity purified goat anti-rabbit IgG (H + L) used with the polyclonal anti-CD3, and fluorescein (FITC)-conjugated affinity purified donkey anti-goat IgG (H + L) and normal donkey serum for use with the anti-pre-TCRα antibody. Purchased from Molecular Probes were: Alexa Fluor 488-conjugated goat anti-mouse IgG1 (used with anti-CD10, anti-tubulin and anti-HIV-1p24), Alexa Fluor 488-conjugated IgG2b (used with anti-CD11b), Alexa Fluor 555-conjugated goat anti-mouse IgG1 (used with anti-tubulin and anti-HIV-1p24) and Alexa Fluor 555-conjugated goat anti-mouse IgG2b (used with CD11b). Antibody dilutions used were those recommended by the supplier or determined by testing.

Cells were cultured in 35 mm poly-D-lysine-coated glass bottom microwell dishes (#P35GC-0-14-C from MatTek Corp). EDTA-recovered macrophages harvested at day 28–31 of culture were seeded at ∼1x10^5^/well in RPMI-20% and cultured as described above, with or without addition of IL-2. In some experiments, macrophages were recovered by adherence from nonadherent populations harvested from primary flask cultures at day 10 and beyond. In these cases, 2–3×10^5^ harvested cells in 50–100 µl of RPMI-20% were applied to the glass portion of the dishes. Several hours later, when the macrophages were well attached, the dishes were washed to remove nonadherent cells, RPMI-20% added and the cells cultured for various times. To harvest the dishes for staining, the medium was removed and 4% paraformaldehyde was added without prior washing. Plates were incubated with fixative for 30 minutes at RT, washed with DPBS and either stained immediately, or stored at 4°C with DPBS.

The staining procedure for all studies except the pre-TCRα and BrdU labeling experiments was as follows: blocking was performed by incubation with 5% goat serum in DPBS containing 0.3% Triton X-100 for at least 1 hour with rocking. The blocking solution was then removed, and primary antibody(ies) diluted with DPBS containing 2% goat serum and 0.3% Triton X-100 (PBS diluent) added. (Typically, because two primary mouse monoclonal antibodies with different subclass specificities were used, or two primaries derived from different species, it was possible to apply both at the same time.) Dishes were incubated overnight at 4°C with slow rocking. Following extensive washing, secondary antibody(ies) diluted in PBS diluent was added, and the dishes incubated 2–6 hours at RT with slow rocking. For counterstaining, dishes were incubated at RT for 2 hours or more with TOTO-3 (diluted 1∶2500–1∶5000 with DPBS), washed, then a glass coverslip applied using 17% Mowiol in DPBS. The dishes were examined using an LSM 510 confocal laser scanning microscope.

For the pre-TCRα experiments, blocking was performed as above, but using donkey serum, and dishes were incubated overnight with anti-pre-Tα antibody. Following washing, the FITC-labeled donkey anti-goat secondary antibody was applied with incubation for 5–6 hours, followed by washing, then blocking with goat serum, and incubation overnight with anti-CD11b antibody. The remaining steps were carried out as described above, using Alex-Fluor 555-conjugated goat anti-mouse IgG2b secondary antibody and TOTO-3.

### Time-lapse Videography

Living macrophage cultures were filmed using an Amscope digital camera (model #MD600E) attached to an Olympus CK2 inverted microscope. The movies were viewed using VLC software, and snapshots of individual images were captured using Grab software (Apple).

### BrdU Labeling Experiments

For evaluation using fluorescence microscopy, macrophage cultures were established and maintained in 24-well plates as described above. Bromodeoxyuridine (BrdU), at a final concentration of 50 µM, was added at days 5, 7, 10 or 14 of culture. Cells were incubated with BrdU for 18–48 hours. Fixation was performed using 70% ethanol for 30 minutes, followed by a PBS wash, incubation with 0.5% Triton X-100 in PBS for 10 minutes to permeabilize the cells, and then a 1 hour incubation with 2N HCl to denature the DNA. The wells were then washed twice using PBS pH 8.0 and incubated for 2 hours with Alexa Fluor 488-conjugated mouse anti-BrdU (BD Biosciences) diluted 1∶8 or FITC-conjugated mouse anti-BrdU (BD Biosciences) diluted 1∶4, in DPBS supplemented with 0.5% Tween 20. Following 3 washes in PBS, the cells were counterstained by incubation with 2.8 µM DAPI (Molecular Probes.) for 10 minutes, washed and examined using a Zeiss Axio Observer Z1 microscope.

For evaluation using confocal microscopy, nonadherent cells from HIV-1-infected and uninfected macrophage cultures were harvested at various times, transferred to 35 mm poly-D-lysine-coated glass bottom microwell dishes, and cultured for an additional 2–4 days. BrdU, at a final concentration of 50 µM, was then added and the dishes incubated for 18–48 hours. Fixation was performed using 4% paraformaldehyde for 30 minutes at room temperature (RT), and followed by three washes using PBS. For immunostaining of BrdU-labeled cells, blocking and initial permeabilization was performed by incubation for 30–60′ at RT in PBS containing 5% goat serum and 0.5% Triton X-100. Following washing, primary antibody was applied (anti-tubulin or anti-CD11b diluted in PBS containing 2–3% goat serum and 0.5% Tween 20) and the dishes incubated overnight at 4°C. After washing, secondary antibody diluted in PBS containing 2–3% goat serum and 0.5% Tween 20 was applied, and the dishes incubated 1–2 hours at RT, then washed. Prior to anti-BrdU staining, the cells were again treated with 4% paraformaldehyde for 30′ at RT, then washed and incubated for 30′ with 2N HCl diluted in DPBS. Following additional washes, the Alexa Fluor 488-conjugated mouse anti-BrdU antibody diluted in DPBS containing 0.5% Tween 20 was added, and the dishes incubated overnight at RT. Counterstaining with TOTO-3 was performed as above.

### Real-time PCR Assays for Pre-TCRα, RAG1 and CD10

These were performed using the RT^2^ qPCR Primer assays offered by SABiosciences. The catalog numbers for these reagents are as follows: for pre-TCRα, catalog # PPH06312E, for RAG1, catalog # PPH09892A, and for CD10, catalog # PPH00422E. The assays were performed according to manufacturer’s specifications. GAPDH was used as a reference gene for normalization. The reference gene reactions were carried out using the SABiosciences GAPDH RT^2^ qPCR Primer assay (catalog #PPH00150E). RNA was purified using the RNeasy mini kit from Qiagen (catalog #74106), and cDNA was prepared using the RT^2^ Easy first strand synthesis kit from Qiagen (catalog # 330401). Reactions were performed in duplicate and evaluated using the ABI Prism 7000 real-time PCR instrument. All reactions pertaining to a given sample were run on the same plate. Mean values and percent difference between duplicates were determined. For all experiments, the average percent difference between duplicates was less than 1.3% (1.3% and 0.5% for pre-TCRα, 0.9% and 0.4% for RAG1, and 0.7% and 0.6% for CD10). We routinely obtain such low levels of variability between duplicates and for this reason, we believe it unnecessary to use more replicates per sample, especially considering the expense of these kinds of experiments.

### Real-time PCR for Detection of TREC

A molecular construct was developed to provide for TREC copy number quantitation. A 408 bp fragment of DNA containing the δRec-ψJα signal-joint break point was amplified from human uncultured PBMC DNA, and inserted into the pCR2.1-TOPO vector (Invitrogen). The primers used for this were: 5′-AAAGAGGGCAGCCCTCTCCAAGGCAAAA-3′ (sense) and 5′-ACTTCCATCGCAATTCAGGACTCACTT-3′ (antisense) [82]. DNA was purified from cultured cells using the DNeasy Blood & Tissue Kit (Qiagen catalog # 69506). The PCR reactions were performed in a total volume of 25 µl, that contained 12.5 µl of SYBR Green/ROX master mix (SABiosciences), 10.5 µl of water, 0.5 µl of each primer, and 1 µl of DNA. The primers used in these reactions were: 5′-CGTGAGAACGGTGAATGAAGAGCAGACA-3′ (sense) and 5′-CATCCCTTTCAACCATGCTGACACCTCT-3′.(antisense) [82]. Samples from the same experiment were run in duplicate on the same plate, along with a dilution series of TREC plasmid DNA, and positive and negative control samples. DNA from thymus, and DNA from the glioblastoma cell line U138MG, were used as positive and negative controls, respectively, Genomic and plasmid DNA were handled in separate rooms, and care was taken to insure no contamination of reagents and assay materials with plasmid DNA. The reactions and analyses were carried out using an ABI Prism 7000 real-time PCR instrument. Values were extrapolated to TREC copy number per µg DNA.

### Sequencing of TREC PCR Products

To confirm the TREC identity of products detected using real-time PCR, we sequenced a few samples identified as TREC positive. Nested PCR was performed using as outer primers those described above for generation of the TREC plasmid, and as inner primers, those used in the TREC real-time PCR assay. The second round reactions were gel purified and sequenced using an ABI 3130 instrument. Sequence alignment was performed using the DNASTAR software.

### Real-time PCR for Detection of HIV-1 Copy Number

CD3 microbeads (Miltenyi Biotec) were used to separate nonadherent cells harvested from HIV-1 pSF162R3- and HIV-1 Ba-L-infected macrophage cultures at days 5 and 8 post infection, respectively. Aliquots of the separated populations were immunostained for T-cell and macrophage markers and analyzed using 4-color flow cytometry. HIV-1 expression in pSF162R3-infected cells was determined by EGFP expression, and expression in Ba-L-infected cells was determined by downregulation of CD4 on CD3+ cells lacking markers of other lineages. The percentage of CD3+CD4+ and CD3-CD14+ cells within their respective bead-separated populations was calculated from flow cytometry data.

The forward primer (RTF) 5′-TGGGTACCAGCACACAAAGG-3′, reverse primer (RTR) 5′-ATCACTAGCCATTGCTCTCCAAT-3′ and MGB probe (RTProbe) 5′-VIC-ATTGGAGGAAATGAAC-3′ were used for determining viral copy number. These correspond to a region of HIV-1 pol (reverse transcriptase), nucleotides 3698–3850 of HXB2. The cycling program consisted of incubation at 50°C for 2 min followed by initial denaturation at 95°C for 10 min, then 40 cycles of 95°C for 15 sec, 60°C for 1 min. Reactions were carried out in 25 µl volumes containing 22.5 pmoles of each primer and 6.25 pmoles of probe. Reactions were performed in replicates and evaluated using the ABI Prism 7000 real-time PCR instrument. ACH2 cell lysates of known HIV-1 copy number were used as standards. Uninfected cell DNA and reagent only negative controls were included. The number of CD3+CD4+ and CD3-CD14+ cells harboring HIV-1 genome was normalized to 100% and compared to the number expressing virus as determined by flow cytometry.

## Supporting Information

Figure S1 **Development of elongated cellular processes in response to IL-2.** Shown here is a secondary culture established from uninfected EDTA-recovered macrophages harvested at day 31. The replated cells were cultured for 2 days, and then washed extensively, prior to treatment with IL-2. The photograph was taken on day 2 of IL-2 exposure. Cells with long thin extended processes are apparent (black arrows). This response is an early step towards development of the nurse macrophage microenvironment. Also apparent here is an epithelioid multinucleated cell (white arrow). Magnification; x200.
(TIF)

Figure S2 **Development of nurse macrophages from CD14+ PBMC.** CD14+ cells were selected from healthy donor PBMC using CD14 microbeads and the Miltenyi cell separation system, and then cultured as described in the Materials and Methods. In this experiment, IL-2 was added on day 10 of culture, and the cells were photographed on day 30 following addition of IL-2. Large, epithelioid macrophages, complex multicellular structures, and numerous small cells are apparent. Many of the small cells are nonadherent; their CD4+ T-cell phenotype was confirmed by flow cytometry. Magnification; x100.
(TIF)

Figure S3 **Low abundance populations in EDTA-recovered IL-2-treated macrophage cultures.** Primary macrophage cultures were subjected to EDTA treatment on day 28 of culture. The recovered cells were reseeded into 24-well plates at 2.7×10^5^/well, cultured for 2 days, then washed and treated with IL-2. (A) Detection of CD4-CD8+ cells (blue dots, 0.74% of total) and CD4+CD8+ cells (red dots, 0.31% of total) on day 21 of IL-2 treatment. Note that the 4/8 dual positive cells are larger than the CD4-CD8+ cells. (B) Detection of CD34+ cells at 112 hours and 15 days of IL-2 exposure (large black dots, 0.25% of total at both time points shown). CD8+ and CD34+ cells were identified on their respective side scatter (SSC) plots.
(TIF)

Figure S4 **Development of T-cells within nurse macrophages.** (This is a gallery view of Figure 2C.) Deposits of free CD3 antigen are apparent (green arrow). CD3+ cells can be seen deep within the macrophage, as well as being released at its surface (white arrow). Fluorochromes: CD3 (Cy2) and CD68 (RRX). Size bar (upper left image) 20 µm, is identical for each picture.
(TIF)

Figure S5 **Response of sorted CD2+CD3+CD16+CD32+ cells to IL-2.** Nonadherent cells were harvested from primary macrophage cultures at day 15 (donor 895) or day 14 (donor 898) of culture, immunostained, and sorted for quadruple expression of CD2, CD3, CD16 and CD32 using a FACSAria cell sorter. The sorted populations were seeded into V-bottom 96-well plates at a density of 80,000–100,000 per well. Owing to the small numbers of sorted cells recovered, only 2 wells were prepared per population. In this experiment, IL-2 was added to one well, and PHA was added to the other. The PHA-exposed cells did not survive beyond day 3, and are not included in these analyses. For each donor, the upper panels show flow cytometry results confirming CD2, CD3, CD16 and CD32 expression on the sorted populations, and the lower panels demonstrate loss of CD16 and CD32 expression on the sorted cells following a 17-day exposure to IL-2. Panels on the left indicate the gates (encircled) used for each set of plots to the right. Bottom series: Photographs of sorted, IL-2-treated donor 895 cells at day 17 of IL-2 treatment. The center and right panels show Wright’s-Giemsa-stained cells harvested from the colony shown in the left panel. A mitotic cell can be seen in the right panel (arrow). These demonstrate that the sorted cells proliferated in reponse to IL-2. Too few sorted cells were available for accurate quantitation of cell numbers over time using counting, or other methods. Size bars: center panel, 20 µm, and right panel, 10 µm. Additional experiments were performed with cells harvested and sorted at day 21 and day 22 of culture. These yielded similar results.
(TIF)

Figure S6 **Sequence alignment of δRec-ψJα signal joints.** (A) Agarose gel electrophoresis of TREC PCR products used for sequencing. Shown are the second round products of nested PCR separated on a 2% agarose gel. The bands from this gel were excised, and the DNA purified and used in sequencing reactions. Lanes: (1) pool of adherent and nonadhererent cells harvested from replated macrophages at day 12 of IL-2 treatment; primary cultures were established from CD3-depleted normal donor 895 PBMC; (2) nonadherent cells from replated macrophages at day 13 of IL-2 treatment; primary cultures were established from CD3-depleted normal donor 895; (3) loosely adherent cells from replated macrophages at day 13 of IL-2 treatment; primary cultures were established from CD3-depleted normal donor 895; (8) unfractionated normal human thymus; (9) pool of adherent and nonadherent cells from replated macrophages from normal donor 225 at day 9 of IL-2 exposure; (10) adherent cells harvested from replated macrophages from normal donor 914 at day 7 of IL-2 exposure; (11) adherent cells harvested from replated macrophages from normal donor 914 at day 10 of IL-2 exposure. (12) TREC plasmid clone. (M) 50 bp DNA ladder. TREC copy numbers for the samples in lanes 2, 3, 8, 10 and 11 are shown in Table 2. (B) Alignment of sequences obtained from the PCR products shown in (A). RSS indicates the conserved nonamer Recombination Signal Sequence important for recognition by VDJ recombinases during VDJ rearrangement.
(TIF)

Figure S7 **Quiescent and replicating DNA within the same macrophage.** These are additional images of the cell shown in Figure 6 J–M. (A) Gallery view. DNA replication, as evidenced by colocalization with cdc6 expression, is proceeding within the right side of the cell (white arrow), while the nucleus on the left is quiescent. (B) Enlargement of the replicating DNA (right side of cell), which illustrates cdc6 expression in association with the globular DNA structures. Fluorochromes: Tubulin (Alexa Fluor 488) and cdc6 (Cy3). Scale bars = 10 µm.
(TIF)

Figure S8 **An HIV-1-expressing small monocytoid cell budding from a nurse macrophage.** (This is a gallery view of Figure 8A.) Colocalization of HIV-1p24 with CD36 can be seen throughout the macrophage, and concentrated within the center of the cell (yellow color). Colocalization is also apparent in the small cell budding from the center of the macrophage, indicating that it is both expressing HIV-1, and belongs to the macrophage lineage (CD36+). As illustrated here, CD36 is not expressed on T-lymphocytes. Neither the CD3+ cells developing within the nurse macrophage, nor those outside of it, are expressing HIV-1. Culture was infected with HIV-1 5E14BM, an isolate recovered from bone marrow, and photographed on ∼day 21 of infection. Fluorochromes; HIV-1p24 (Alexa 488), CD3 (Cy5) and CD36 (Cy3). Size bar (shown in upper left image) is the same for each picture of the series, 20 µm.
(TIF)
