# Supplementary figures and images for: De novo Generation of Cells within Human Nurse Macrophages and Consequences following HIV-1 Infection
Source: PLoS One. 2012 Jul 18;7(7):e40139. doi: 10.1371/journal.pone.0040139 (PMC3399863; doi:10.1371/journal.pone.0040139)

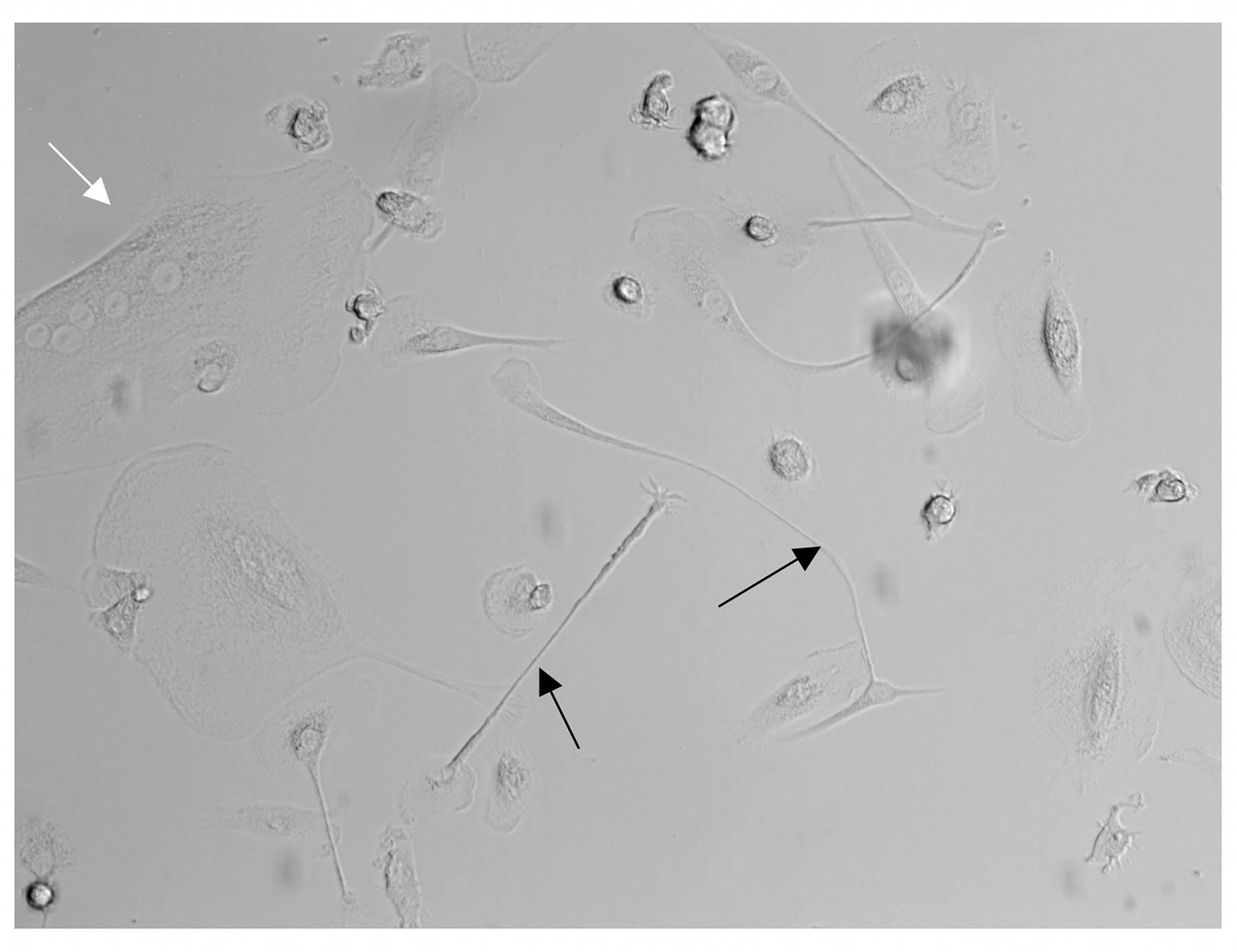

Supplement: Figure S1 — Development of elongated cellular processes in response to IL-2. Shown here is a secondary culture established from uninfected EDTA-recovered macrophages harvested at day 31. The replated cells were cultured for 2 days, and then washed extensively, prior to treatment with IL-2. The photograph was taken on day 2 of IL-2 exposure. Cells with long thin extended processes are apparent (black arrows). This response is an early step towards development of the nurse macrophage microenvironment. Also apparent here is an epithelioid multinucleated cell (white arrow). Magnification; x200. (TIF) [file pone.0040139.s001.tif]

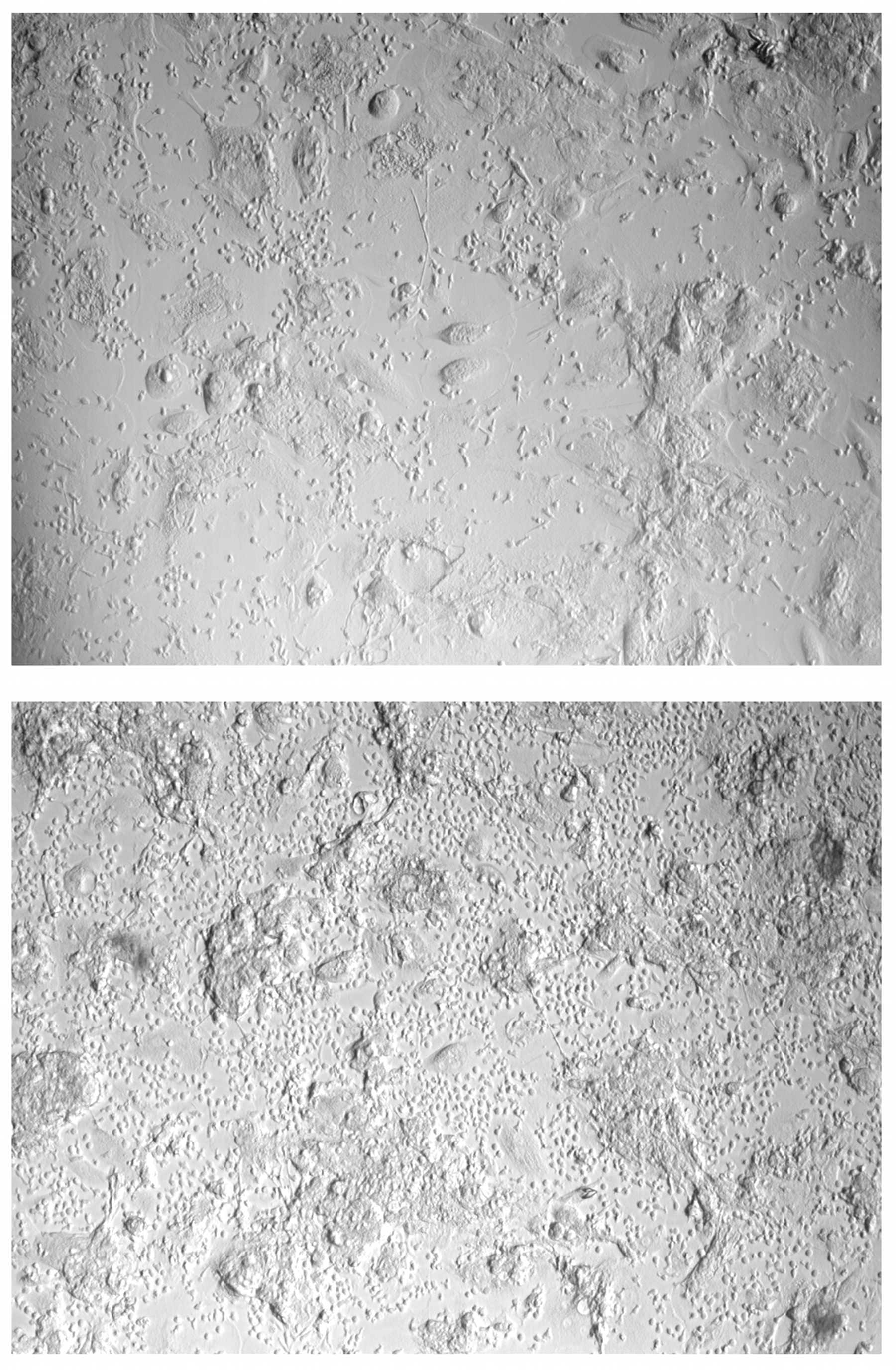

Supplement: Figure S2 — Development of nurse macrophages from CD14+ PBMC. CD14+ cells were selected from healthy donor PBMC using CD14 microbeads and the Miltenyi cell separation system, and then cultured as described in the Materials and Methods. In this experiment, IL-2 was added on day 10 of culture, and the cells were photographed on day 30 following addition of IL-2. Large, epithelioid macrophages, complex multicellular structures, and numerous small cells are apparent. Many of the small cells are nonadherent; their CD4+ T-cell phenotype was confirmed by flow cytometry. Magnification; x100. (TIF) [file pone.0040139.s002.tif]

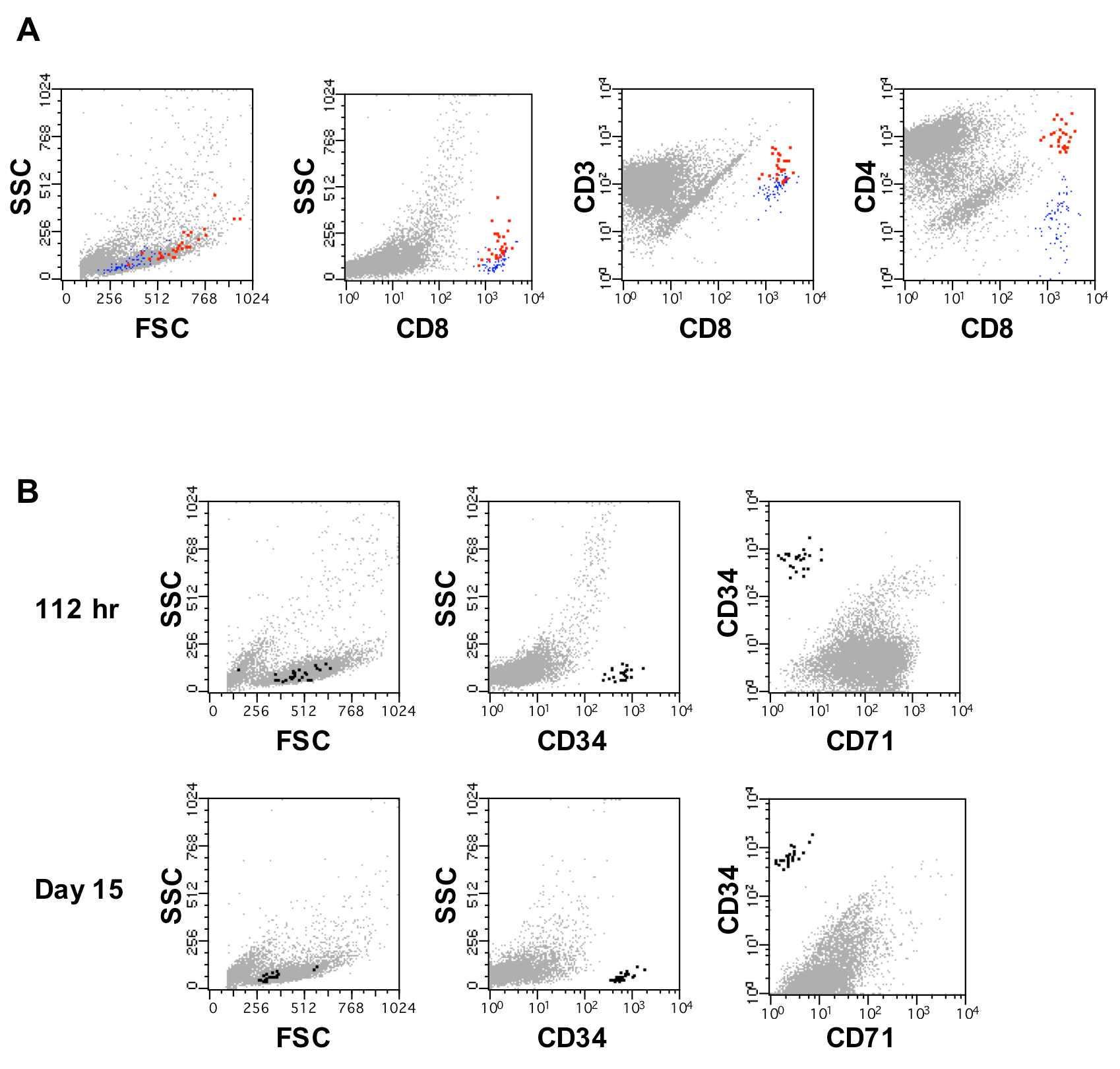

Supplement: Figure S3 — Low abundance populations in EDTA-recovered IL-2-treated macrophage cultures. Primary macrophage cultures were subjected to EDTA treatment on day 28 of culture. The recovered cells were reseeded into 24-well plates at 2.7×10^5^/well, cultured for 2 days, then washed and treated with IL-2. (A) Detection of CD4-CD8+ cells (blue dots, 0.74% of total) and CD4+CD8+ cells (red dots, 0.31% of total) on day 21 of IL-2 treatment. Note that the 4/8 dual positive cells are larger than the CD4-CD8+ cells. (B) Detection of CD34+ cells at 112 hours and 15 days of IL-2 exposure (large black dots, 0.25% of total at both time points shown). CD8+ and CD34+ cells were identified on their respective side scatter (SSC) plots. (TIF) [file pone.0040139.s003.tif]

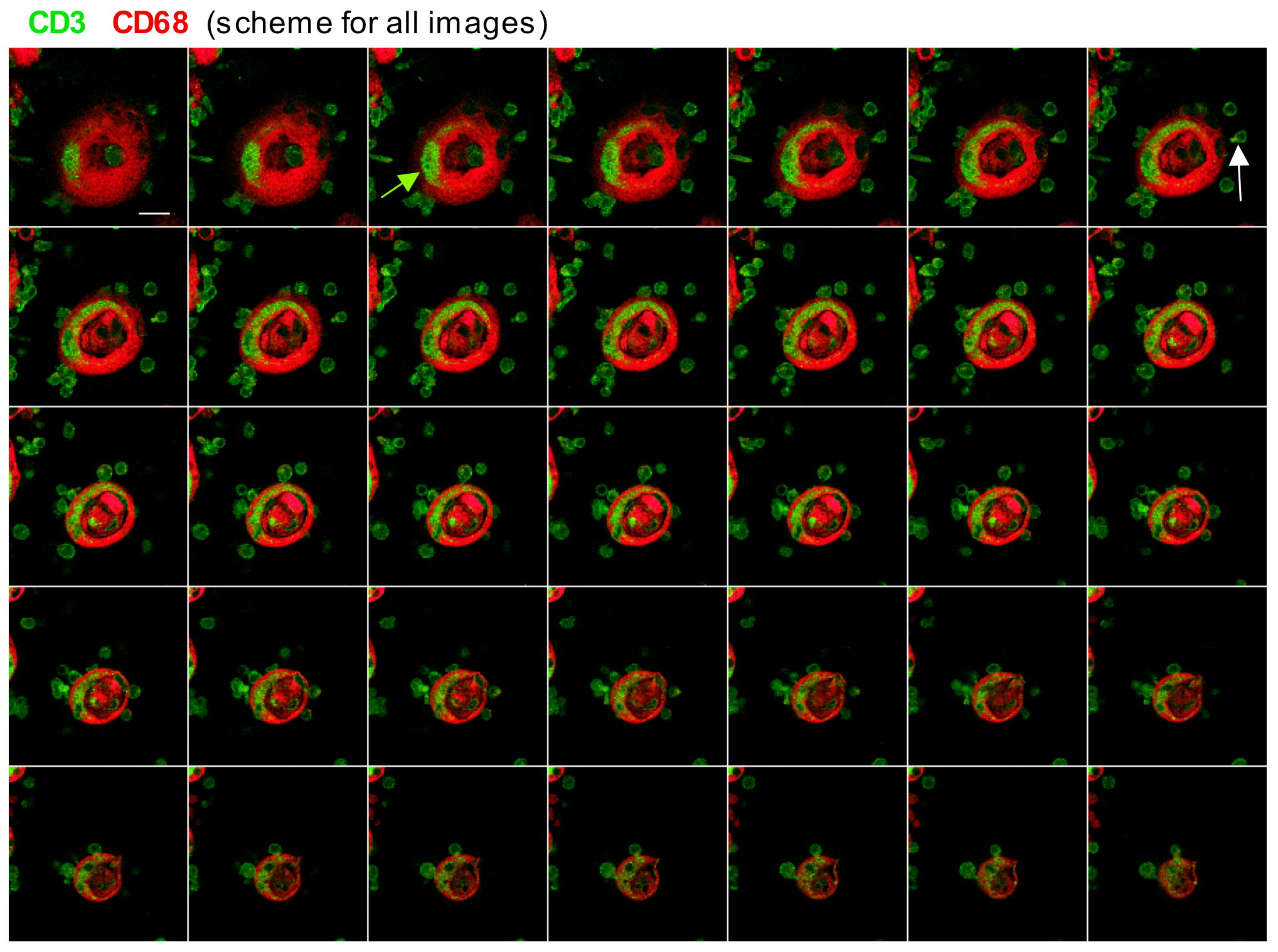

Supplement: Figure S4 — Development of T-cells within nurse macrophages. (This is a gallery view of Figure 2C.) Deposits of free CD3 antigen are apparent (green arrow). CD3+ cells can be seen deep within the macrophage, as well as being released at its surface (white arrow). Fluorochromes: CD3 (Cy2) and CD68 (RRX). Size bar (upper left image) 20 µm, is identical for each picture. (TIF) [file pone.0040139.s004.tif]

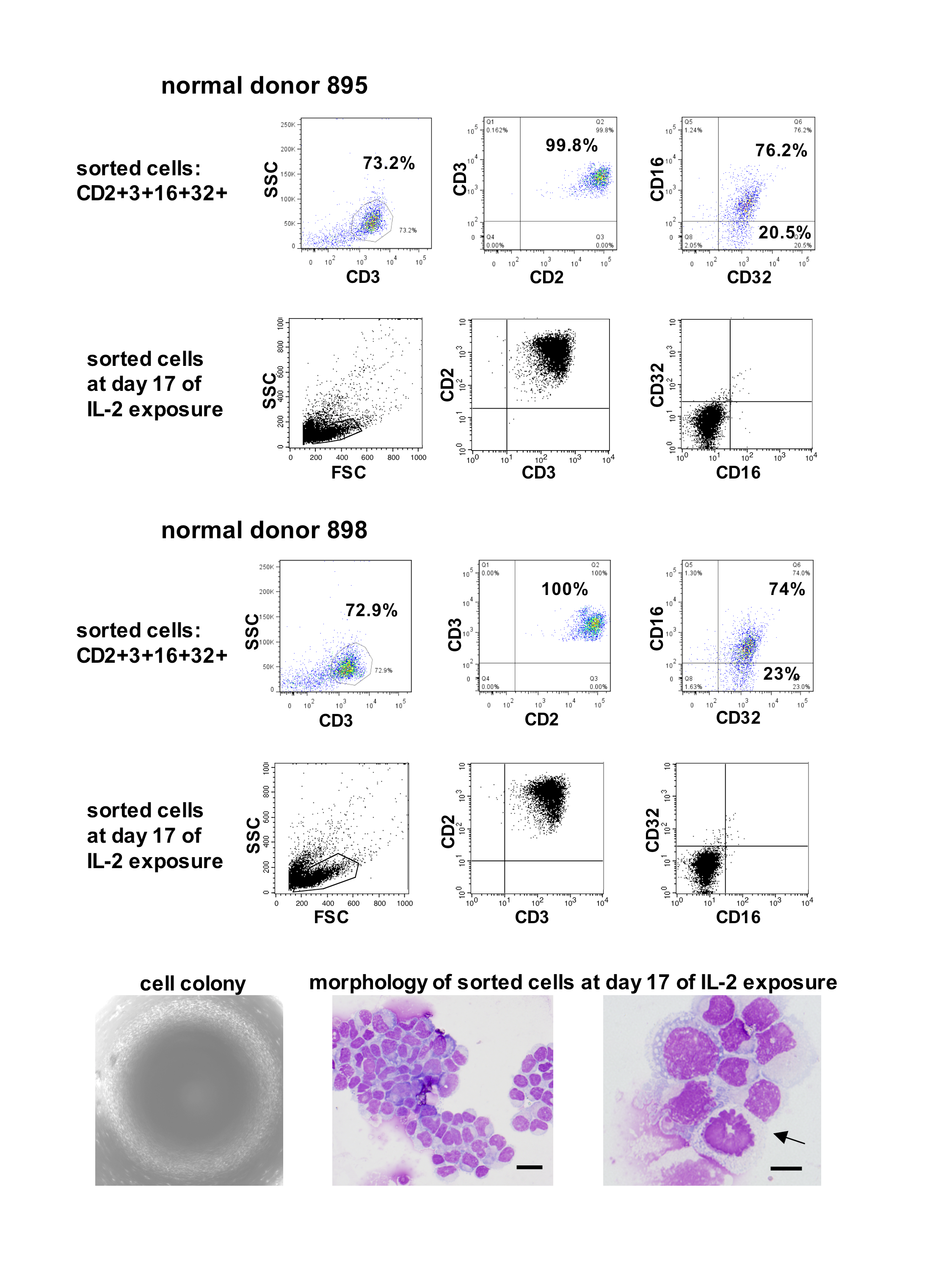

Supplement: Figure S5 — Response of sorted CD2+CD3+CD16+CD32+ cells to IL-2. Nonadherent cells were harvested from primary macrophage cultures at day 15 (donor 895) or day 14 (donor 898) of culture, immunostained, and sorted for quadruple expression of CD2, CD3, CD16 and CD32 using a FACSAria cell sorter. The sorted populations were seeded into V-bottom 96-well plates at a density of 80,000–100,000 per well. Owing to the small numbers of sorted cells recovered, only 2 wells were prepared per population. In this experiment, IL-2 was added to one well, and PHA was added to the other. The PHA-exposed cells did not survive beyond day 3, and are not included in these analyses. For each donor, the upper panels show flow cytometry results confirming CD2, CD3, CD16 and CD32 expression on the sorted populations, and the lower panels demonstrate loss of CD16 and CD32 expression on the sorted cells following a 17-day exposure to IL-2. Panels on the left indicate the gates (encircled) used for each set of plots to the right. Bottom series: Photographs of sorted, IL-2-treated donor 895 cells at day 17 of IL-2 treatment. The center and right panels show Wright’s-Giemsa-stained cells harvested from the colony shown in the left panel. A mitotic cell can be seen in the right panel (arrow). These demonstrate that the sorted cells proliferated in reponse to IL-2. Too few sorted cells were available for accurate quantitation of cell numbers over time using counting, or other methods. Size bars: center panel, 20 µm, and right panel, 10 µm. Additional experiments were performed with cells harvested and sorted at day 21 and day 22 of culture. These yielded similar results. (TIF) [file pone.0040139.s005.tif]

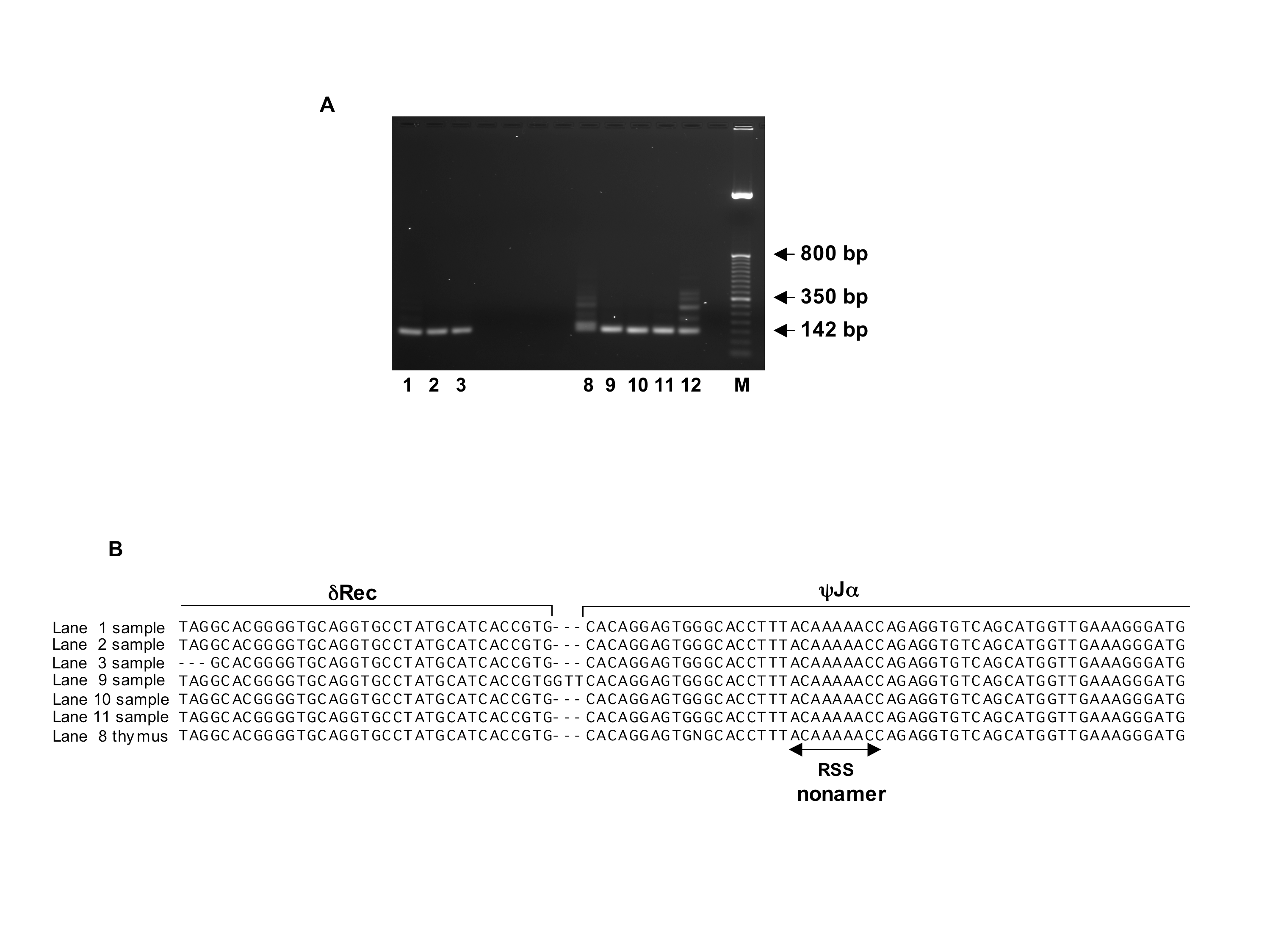

Supplement: Figure S6 — Sequence alignment of δRec-ψJα signal joints. (A) Agarose gel electrophoresis of TREC PCR products used for sequencing. Shown are the second round products of nested PCR separated on a 2% agarose gel. The bands from this gel were excised, and the DNA purified and used in sequencing reactions. Lanes: (1) pool of adherent and nonadhererent cells harvested from replated macrophages at day 12 of IL-2 treatment; primary cultures were established from CD3-depleted normal donor 895 PBMC; (2) nonadherent cells from replated macrophages at day 13 of IL-2 treatment; primary cultures were established from CD3-depleted normal donor 895; (3) loosely adherent cells from replated macrophages at day 13 of IL-2 treatment; primary cultures were established from CD3-depleted normal donor 895; (8) unfractionated normal human thymus; (9) pool of adherent and nonadherent cells from replated macrophages from normal donor 225 at day 9 of IL-2 exposure; (10) adherent cells harvested from replated macrophages from normal donor 914 at day 7 of IL-2 exposure; (11) adherent cells harvested from replated macrophages from normal donor 914 at day 10 of IL-2 exposure. (12) TREC plasmid clone. (M) 50 bp DNA ladder. TREC copy numbers for the samples in lanes 2, 3, 8, 10 and 11 are shown in Table 2. (B) Alignment of sequences obtained from the PCR products shown in (A). RSS indicates the conserved nonamer Recombination Signal Sequence important for recognition by VDJ recombinases during VDJ rearrangement. (TIF) [file pone.0040139.s006.tif]

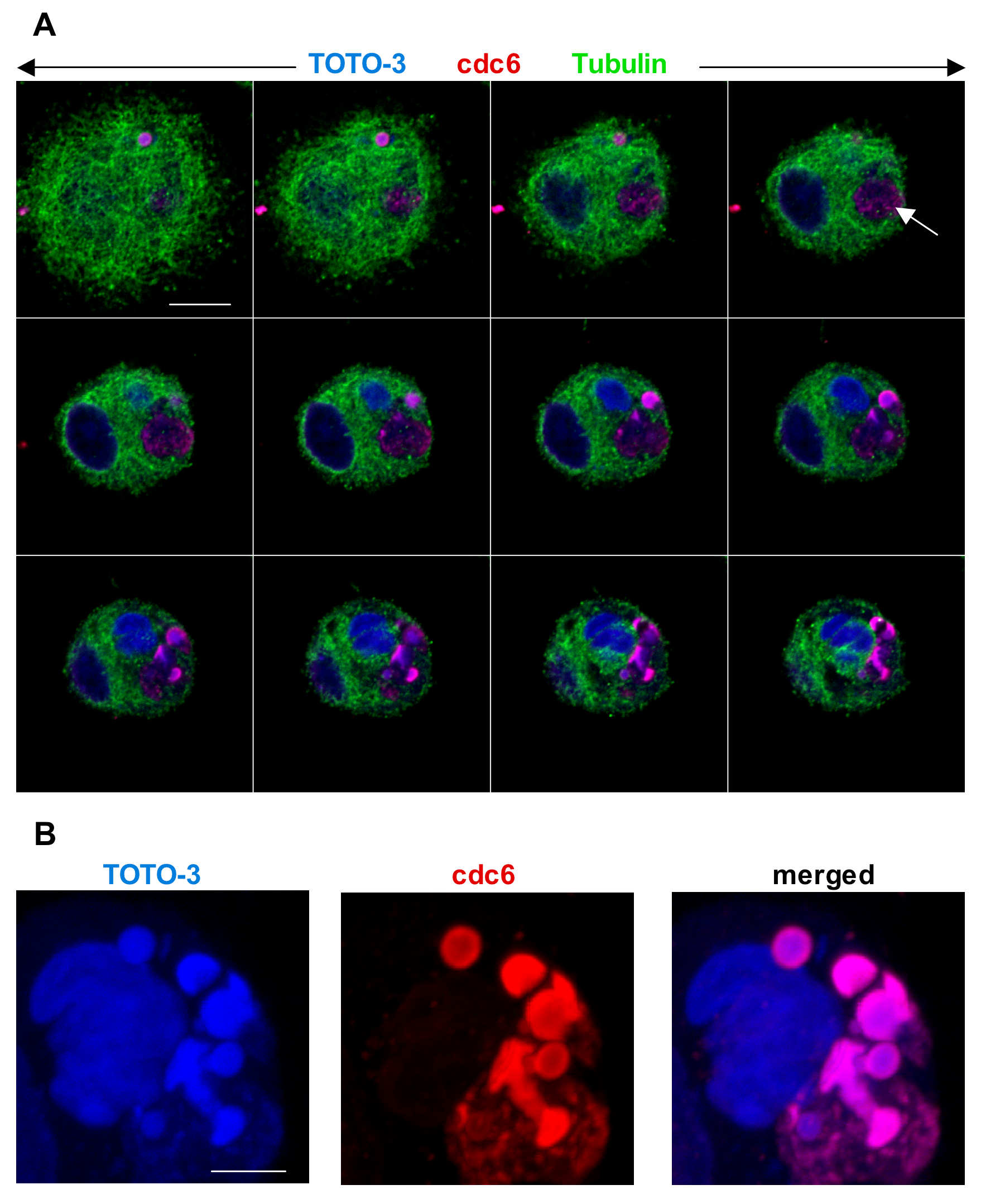

Supplement: Figure S7 — Quiescent and replicating DNA within the same macrophage. These are additional images of the cell shown in Figure 6 J–M. (A) Gallery view. DNA replication, as evidenced by colocalization with cdc6 expression, is proceeding within the right side of the cell (white arrow), while the nucleus on the left is quiescent. (B) Enlargement of the replicating DNA (right side of cell), which illustrates cdc6 expression in association with the globular DNA structures. Fluorochromes: Tubulin (Alexa Fluor 488) and cdc6 (Cy3). Scale bars = 10 µm. (TIF) [file pone.0040139.s007.tif]

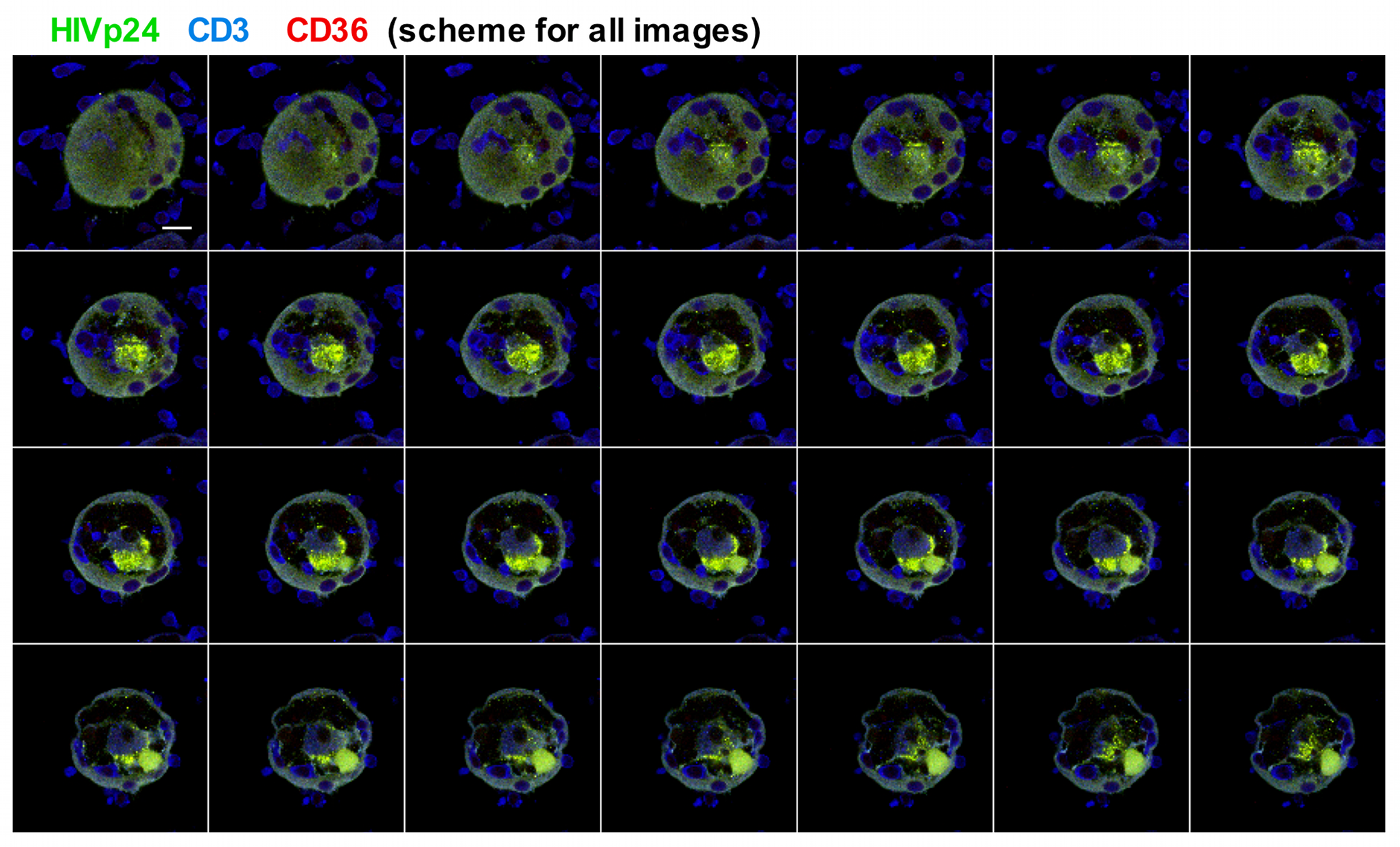

Supplement: Figure S8 — An HIV-1-expressing small monocytoid cell budding from a nurse macrophage. (This is a gallery view of Figure 8A.) Colocalization of HIV-1p24 with CD36 can be seen throughout the macrophage, and concentrated within the center of the cell (yellow color). Colocalization is also apparent in the small cell budding from the center of the macrophage, indicating that it is both expressing HIV-1, and belongs to the macrophage lineage (CD36+). As illustrated here, CD36 is not expressed on T-lymphocytes. Neither the CD3+ cells developing within the nurse macrophage, nor those outside of it, are expressing HIV-1. Culture was infected with HIV-1 5E14BM, an isolate recovered from bone marrow, and photographed on ∼day 21 of infection. Fluorochromes; HIV-1p24 (Alexa 488), CD3 (Cy5) and CD36 (Cy3). Size bar (shown in upper left image) is the same for each picture of the series, 20 µm. (TIF) [file pone.0040139.s008.tif]
